# Integrated Phytochemical Analysis Based on UPLC–MS/MS and Network Pharmacology Approaches to Explore the Effect of *Odontites vulgaris* Moench on Rheumatoid Arthritis

**DOI:** 10.3389/fphar.2021.707687

**Published:** 2021-08-30

**Authors:** Mingyue Ji, Congcong Wang, Tieyi Yang, Xiangxi Meng, Xiaoqin Wang, Minhui Li

**Affiliations:** ^1^ Inner Mongolia Key Laboratory of Characteristic Geoherbs Resources Protection and Utilization, Baotou Medical College, Baotou, China; ^2^ Trauma Orthopedic, The Second Affiliated Hospital of Inner Mongolia Medical University, Hohhot, China; ^3^ Department of Pharmacy, Inner Mongolia Medical University, Hohhot, China; ^4^ Inner Mongolia Institute of Traditional Chinese Medicine, Hohhot, China; ^5^ Key Laboratory of Resourceology of Chinese Medicinal Materials, Baotou, China; ^6^ Inner Mongolia Engineering Research Center of The Planting and Development of Astragalus Membranaceus of the Geoherbs, Baotou, China

**Keywords:** *Odontites vulgaris*, rheumatoid arthritis, UPLC-MS/MS, network pharmacology, PI3k-Akt signal pathway, RAW 264.7

## Abstract

*Odontites vulgaris* Moench has the effect of clearing away heat, detoxification, dispelling wind, and clearing dampness. In this study, the potential anti-inflammatory compounds of *O. vulgaris* were investigated using ultra-high-performance liquid chromatography–mass spectrometry (UPLC–MS/MS) combined with the network pharmacology approach and further confirmed on an LPS-activated RAW 264.7 macrophage model. Monomer compounds were prepared from the active fraction using modern advanced separation and purification methods. UPLC–Q-Exactive HRMS was used to identify the chemical compounds in the active fractions of *O. vulgaris*. D-mannitol, geniposidic acid, salidroside, shanzhiside methyl ester, eleutheroside B, geniposide, 7,8-dihydroxycoumarin, gardoside methyl ester, arenarioside, vanillic acid, p-hydroxy-cinnamic acid, melampyroside, syringaresinol, tricin, and diosmetin were isolated from *O. vulgaris* for the first time. A compound database of *O. vulgaris* was established based on the existing literature to predict the mechanism of *O. vulgaris* in the treatment of rheumatoid arthritis. The results suggest that the PI3K-Akt pathway mediates *O. vulgaris* and deserves more attention in the treatment of RA. Finally, the anti-rheumatoid arthritis effects of the four target compounds were validated with the decreased levels of NO, TNF-*α*, IL-6 and IL-1*β* in RAW 264.7 macrophage cells treated with LPS. The present study explored the potential targets and signaling pathways of *O. vulgaris* in the treatment of RA, which may help to illustrate the mechanisms involved in the action of *O. vulgaris* and may provide a better understanding of the relationship between *O. vulgaris* and RA. This study provides novel insights into the development of new drugs and utilization of Mongolian traditional Chinese medicine resources.

## Introduction

Rheumatoid arthritis (RA) is a chronic autoimmune disease characterized by persistent synovial hyperplasia, inflammatory infiltration, pannus formation, and bone erosion, which may lead to joint deformity, disability, and death (Li et al., 2019a; Li et al., 2019b; Guo et al., 2020). Thus far, pain relief and reduction of inflammation have been considered the main therapeutic strategies for RA. With a high disability rate, RA poses a serious threat to human health and is a prevalent, globally distributed incurable diseases. Without timely treatment, the incidence of disability in patients with RA is reported to be approximately 70% (Li and Gu, 2020). Non-steroidal anti-inflammatory drugs are commonly used for the treatment of such patients. However, long-term use of these drugs can cause considerable side effects; for example, gastrointestinal mucosal injury and bleeding occur more frequently in patients with long-term use of these drugs than in those with gastrointestinal diseases (Vetal et al., 2013; Li et al., 2018). Biological blockers that suppress inflammation, such as that mediated by anti-tumor necrosis factor-*α*, may lead to a higher risk of infection owing to their role in weakening the immune system (Wang et al., 2020a). The interaction of a drug with an unintended target may be a source of adverse drug reactions and side effects. Therefore, there is an urgent need to find safe and effective drugs for the treatment of RA with fewer side effects than those currently used. In recent years, owing to the low level of ethnopharmacological adverse reactions, Ethnopharmacological Mongolian medicine has attracted attention as an alternative treatment for inflammation (Wang et al., 2020b). These Mongolian medicinal treatments exert anti-inflammatory effects through the NF-κB, MAPK, PI3K-Akt, arachidonic acid metabolism, inflammasome, and other pathways (Mao et al., 2019).

**FIGURE 1:**
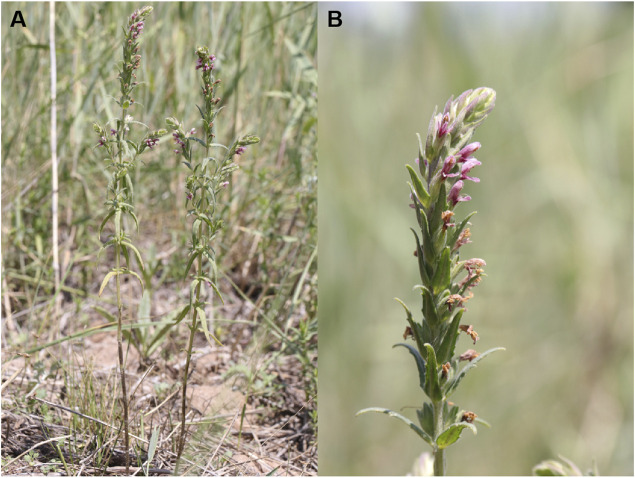
The images of ***Odontites vulgaris* Moench** (**A** and **B**).

**FIGURE 2:**
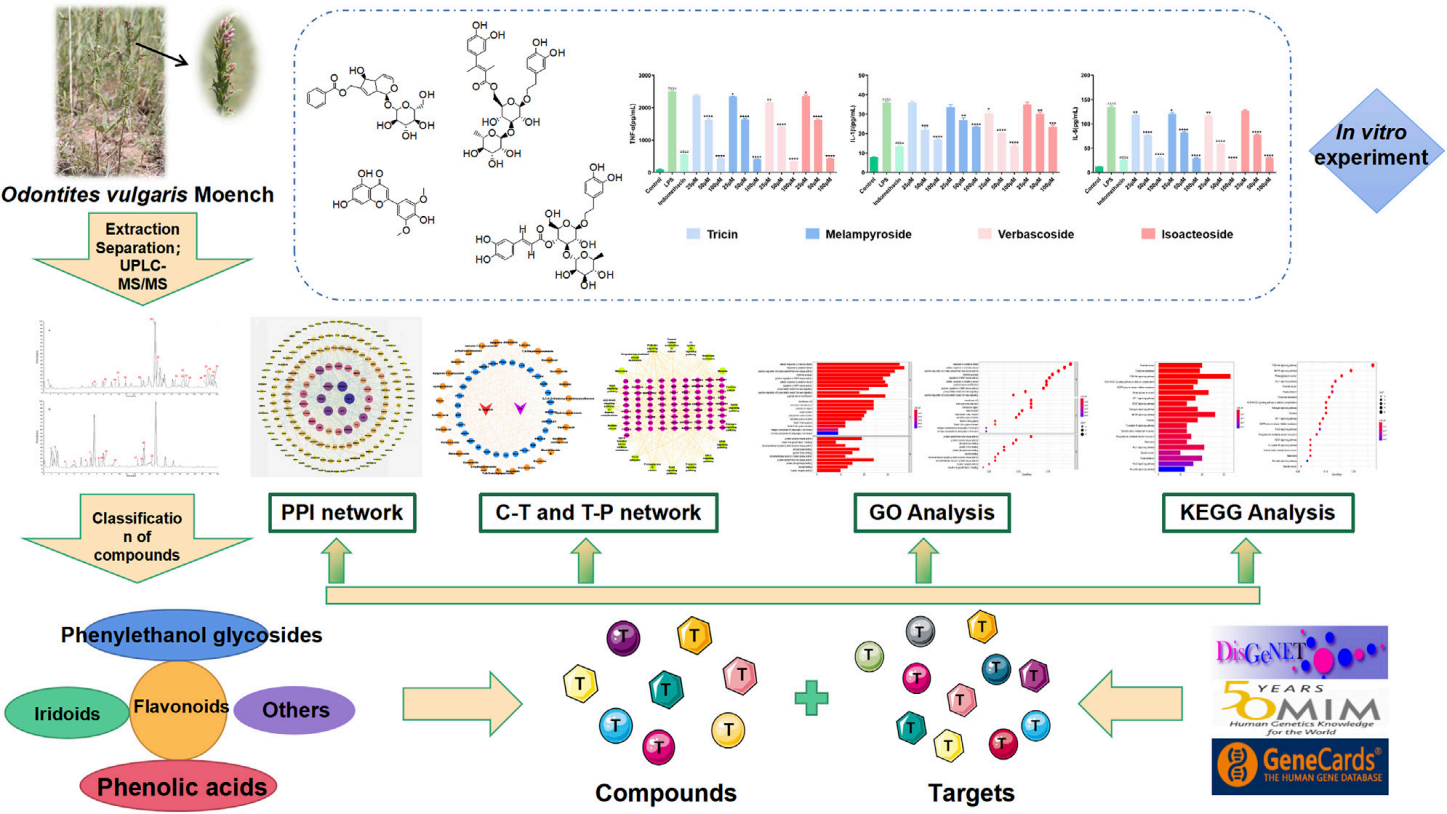
Flow chart of network pharmacological and experimental studies on the effect of *O.* *vulgaris* in the treatment of RA.

In Mongolian medicine, RA is often called “Xieri Wusu” or “Tuolai disease” (Wang et al., 2018) and is a “Taolai” disease (Bai et al., 2015). “Basaga,” with its name derived from the transliteration of a Sanskrit word, is often used in Mongolian medicine with remarkable curative effects, such as cooling blood, reducing itching, detoxification, and diuresis (Zhao et al., 2017). According to traditional Chinese medicine, RA is a disease caused by wind, cold, dampness, heat, and other evil gases that close and obstruct meridians and collateralization, leading to pain in the limbs, bones, and joints caused by poor flexion and extension, stiffness, and deformation (Liu et al., 2015). *Odontites vulgaris* Moench (Figure 1) is a commonly used Mongolian medicine variety of “Basaga.” It helps in clearing away heat, detoxification, dispelling wind, and clearing dampness. The primary compounds present in *O. vulgaris* are phenylethanol glycosides, iridoids, and flavonoids. Some compounds have been shown to have certain anti-inflammatory effects (Gong et al., 2020; Nigro et al., 2020; Wang et al., 2020d; Cicek et al., 2021; Tian et al., 2021). Through interviews with the local populace and hospital personnel in Inner Mongolia, we found that *O. vulgaris* is often used to treat RA; therefore, we conducted an in-depth study on the effects of *O. vulgaris* on RA. A flowchart of this process is shown in Figure 2.

At present, multi-compound, multi-target therapy has been reported to be more effective and less toxic than the use of traditional single-target drugs (Zhang et al., 2013a). Network pharmacology is a new, promising, and low-cost drug development method based on bioinformatics, systems biology, and multi-pharmacology. For Mongolian traditional Chinese medicines with complex compositions and unclear mechanisms of action, especially medicine varieties with weak foundations in research, network pharmacology not only serves as an effective tool for studying the mechanism of action, but also provides a basis for designing *in vivo* and *in vitro* experiments (Zhang et al., 2013a; Zheng et al., 2014; Zhou et al., 2016; Jiang et al., 2019).

To the best of our knowledge, this study is the first to use UPLC–MS/MS technology to study the chemical compounds produced by *O. vulgaris*. We isolated 17 compounds, of which 15 compounds were newly reported. We used network pharmacology combined with computer assisted techniques-molecular docking to predict the target and action pathways of *O. vulgaris* in RA treatment and detected its target compounds from the level of inflammatory factors *in vitro*. This study explored potential targets and signaling pathways for the treatment of RA with *O. vulgaris*, which may help to illustrate the mechanisms of action involved in RA. Moreover, our results can promote the development and utilization of Mongolian traditional Chinese medicine resources.

## Materials and Methods

### Plant Materials

*O. vulgaris* was collected from the Xilinguole League of Inner Mongolia Autonomous Region in 2018 and identified as *Odontites vulgaris* Moench by a specialist. The specimen (specimen number: 152500180629025LY) was kept in the herbarium of Baotou Medical College.

### Chemical Materials

RAW 264.7 cells were purchased from the Cell Bank at the China Academy of Science (Shanghai, China). Fetal bovine serum (FBS), Dulbecco’s modified Eagle’s medium (DMEM), phosphate-buffered saline, penicillin, and streptomycin were purchased from Gibco (New York, United States). Dimethyl sulfoxide (DMSO) and 3-(4,5-Dimethylthiazol-2-yl)-2,5-diphenyltetrazolium bromide (MTT) were purchased from Sangon Biotech (Shanghai, China). Lipopolysaccharides (LPS) and indomethacin were obtained from Sigma-Aldrich (United States). The nitric oxide (NO) kit was purchased from the Nanjing Jiancheng Bioengineering Institute (Nanjing, China). Enzyme-linked immunosorbent assay (ELISA) kits for interleukin-1*β* (IL-1*β*), interleukin**-**6 (IL-6), and tumor necrosis factor-*α* (TNF-*α*) were obtained from Beijing Solarbio Science & Technology Co. Ltd (China). Ultrapure water was obtained from Gen Pure (Thermo Fisher Scientific, Waltham, MA, United States). Acetonitrile (HPLC grade) was purchased from Thermo Fisher Scientific. All reagents were of analytical grade. Reference compounds for the 15 analytes were purchased from the National Institute for the Control of Pharmaceutical and Biological Products (Beijing, China). Purified water was obtained using a Milli-Q system (Millipore, Burlington, MA, United States).

### Extraction and Processing

Whole dried *O.* *vulgaris* (6.04 kg) powder was successively extracted with 70% ethanol; approximately 1.2 kg of the extract was obtained. The extract was dissolved in water and extracted with petroleum ether, ethyl acetate, and n-butanol to obtain 17.56, 73, and 259 g of dry extracts, respectively. The aqueous phase contained 755 g of residue.

### Screening of the Protective Effects of the *O. vulgaris* Extracts on LPS-Induced Inflammation in RAW 264.7 Cells.

#### Cell Culture

RAW 264.7 cells were cultured in DMEM supplemented with 10% FBS and a mixture of penicillin and streptomycin. The cells were incubated at 5% CO_2_ and 37°C.

#### Effect of the Crude Extract of *O. vulgaris* on Cell Viability

Logarithmic phase RAW 264.7 cells seeded in 96-well plates at a density of 1 × 10^5^ cells/ml were cultured at 37°C in a 5% CO_2_ incubator for 24 h. After adherence of the cells to the plates, a series of different concentrations (25, 50, 100, 200, and 400 μg/ml) of the *O.* *vulgaris* extracts (water, n-butanol, ethyl acetate, and petroleum ether) was added. The control group (without the extract) was set at the same time. Five parallel wells were used in each group. After 48 h of culture, 10 μl of the freshly prepared 5 mg/ml MTT solution was added to each well, and the culture was incubated at 37°C for 4 h in the dark. The supernatant was discarded, and 150 µl of dimethyl sulfoxide (DMSO) was added to each well. After oscillation for 10 min, the absorbance (OD 570 nm) of each well was measured using a microplate analyzer (Thermo Scientific Multiskan FC, Thermo Fisher Scientific).

#### Determination of Indomethacin Concentration

Logarithmic phase RAW 264.7 cells seeded in 24-well plates at a density of 5 × 10^5^ cells/ml were placed in a 37°C 5% CO_2_ incubator for 24 h. After adherence to the plate, the cells were randomly divided into the control group, model group (LPS), and positive drug group (indomethacin group). Each group was placed in parallel holes. The cells were cultured with LPS (1 μg/ml) for 4 h, with the positive drug group being administered indomethacin solutions of different concentrations to co-interact with LPS cells for 20 h at the following concentrations: 6.25, 12.5, 25, 50, and 100 µM. The cell supernatant of each group was collected after culture, and NO release in the cell supernatant was detected according to the manufacturer’s instructions. The concentration of NO was determined using sodium nitrite as the control substance, and the 50% inhibition rate (i.e., the IC_50_ value) was calculated according to the concentration of NO. Absorbance at 550 nm was determined using a microplate analyzer.

#### Cell Viability Assay and Protective Effects of the Extracts on LPS-Treated RAW 264.7 Cells

Single-cell suspension RAW 264.7 cells were seeded at a density of 5 × 10^5^ cells/ml in 24-well plates and placed at 37°C in a 5% CO_2_ incubator for 24 h until the logarithmic phase was reached. The group demarcation based on treatment in this study was as follows: control group with normal cell culture for 24 h without any treatment; inflammation model group with cells stimulated using 1 μg/ml LPS for 24 h; negative control group with cells treated using different extracts (alcohol, ethyl acetate, n-butanol, water, and petroleum ether fractions) of *O.* *vulgaris* at 400 μg/ml for 20 h; and the experimental group with cells stimulated with 1 μg/ml LPS for 4 h and different concentrations (12.5, 25, 50, 100, and 200 μg/ml) of *O.* *vulgaris* extracts added to the cells for 20 h. The cell supernatant of each group was collected after culture, and NO release in the cell supernatant was detected according to the Griess test described in the NO kit. Absorbance was measured at 550 nm (Gong et al., 2020).

### Chemical Isolation of Pure Compounds

The n-butanol extract (80 g) was separated on a D101 macroporous resin column (ethanol-water, 70:30), and eight fractions were obtained. Thin-layer chromatography was used to analyze the compounds. Three main fractions (Frs. A1–A3) were ultimately obtained by combining the fractions with similar polarity and compound composition. Separation was carried out on an octadecylsilyl (ODS) column (separation principle: solute polarity difference, 800 g; methanol: H_2_O, 30:70, 60:40, and 90:10; 2.5, 2.5, and 4 L, v/v). After separation and purification, 7.6 mg of compound 1 and 2.4 mg of compound 2 were obtained. Fr. A2 was analyzed using atmospheric pressure silica gel column chromatography and confirmed using Sephadex gel column (Sephadex LH-20) chromatography. We obtained 5.3 mg of compound 3. Fr. A3 was further separated on an ODS column (280 g; methanol: H_2_O, 40:60; 3 L, v/v) into three compounds (B1–B3). Fr. B1 was repeatedly eluted with Sephadex LH-20 (methanol: H_2_O, 45:55) to yield 25.4 mg of compound 4 and 6.4 mg of compound 5. Fr. B2 was separated on a silica gel column (methanol: H_2_O, 5:95–90:10) and eluted with methanol on a Sephadex LH-20 column to obtain 14 mg each of compounds 6 and 7. Compounds 8 (16.6 mg) and 9 (27.3 mg) were obtained from Fr. B3 using preparative HPLC. Compounds 10 (34.9 mg) and 11 (19.8 mg) were obtained from Fr. C4 by repeated elution with Sephadex LH-20 (methanol-water, 60:40).

Ethyl acetate extract (60 g) was prepared using a small pore resin coagulant gel column (MCI; separation principle: gel filtration and reverse-phase distribution, 700 g; ethanol: H_2_O, 30:70, 60:40, and 90:10; 2.5, 2.5, and 3 L, v/v) for chromatographic analysis, and nine fractions (D1–D9) were obtained. Compounds 12 (9 mg) and 13 (10 mg) were obtained using preparative liquid chromatography. Compounds 14 (34.0 mg) and 15 (22 mg) were obtained from Fr. D3–D5 using a silica gel column and a Sephadex LH-20 column. Compounds 16 (20.4 mg) and 17 (18.7 mg) were isolated from Fr. D8–D9 using RP-18, methanol-water gradient elution, Sephadex LH-20, and preparative HPLC.

### UPLC–MS

#### Ultra-high-performance Liquid Chromatography–Mass Spectrometry (UPLC-MS/MS) System

The UPLC high-resolution MS Dionex Thermo Scientific UItimate 3,000 system connected with the Thermo Q Exactive focus machine was used in this study. For the analysis, the extracts of n-butanol and ethyl acetate were redissolved (1 mg/ml) in methanol and the sample was filtered through a 0.22 µm microporous membrane to obtain the test solution, and 10 µl of the filtered solution was injected into the instrument.

#### UPLC–MS/MS System and Operating Conditions

A C18 column (100 × 2.1 mm, 3 μm; Thermo Fisher Scientific) was used in the experiment. The mobile phase consisted of 0.1% formic acid aqueous solution (A), acetonitrile (B), gradient elution, 0–35 min, 95%–5% (A), 5–95% (B). The injection volume was 1 μl, flow rate was 0.3 ml/min, and column temperature was 25°C.

Mass spectrometry conditions were as follows: ion source HESI source, negative ion detection mode; sheath gas pressure, 206.84 kPa; auxiliary gas volume flow, 15.22 L/min; spray voltage, 3.5 kV; ion transmission tube temperature, 320°C; auxiliary gas temperature, 350°C; scanning mode full MS/dd-MS^2^, full MS resolution 70,000; and dd-MS^2^ resolution 17,500, scanning range m/z 100–1,500. For MS/MS, the collision energies were 20, 40, and 60 eV. All MS spectra were collected using the Xcalibur 3.0 software of the UPLC–Q-Exactive quadrupole electrostatic field orbit trap high-resolution mass spectrometer.

#### Data Processing

After the sample solution was analyzed and detected according to the above mass spectrum conditions, the Qualbrowser feature of the Xcalibur 3.0 software was used to complete data acquisition and processing, including the extraction of the ion chromatogram and fragmentation behavior information. The mass error of each main fragment ion was within 5 ppm. Full-scan mode high-resolution mass spectrometry data were analyzed using the Mass Frontier 7.0 software, and the possible structure formulae were deduced. The actual molecular weights of the detected compounds were then matched with the theoretical values. The Discoverer 2.1 (including mzCloud, Chinese medicine compound database, Chemspider, and Sci finder) database was used to search for the compounds, match the first and second mass spectra of the compounds, analyze the pyrolysis law, and identify the compounds.

### Network Pharmacology

#### Prediction of *O. vulgaris* Compound Information

The compound database was established through literature retrieval and compound identification in Chinese and English databases (CNKI, Wanfang, PubMed). Compound information was collated in the PubMed database (Hao et al., 2013). The PubChem database constantly adds new data, and the SMILES or chemical structure formulae can be uploaded to the Swiss target prediction database (Daina et al., 2019).

#### Prediction of *O. vulgaris* Targets for RA Treatment

Using “rheumatoid arthritis” as the keyword, RA-related targets were identified in the OMIM, GeneCards, and DisgeNet databases. The TBtools software was used to match the targets related to the chemical compounds of *O.* *vulgaris* with the targets of RA. Additionally, a Venn diagram was drawn to obtain the potential therapeutic targets of the compounds of *O.* *vulgaris* for RA.

#### Construction and Analysis of the Protein-Protein Interaction Networks of the Target Proteins

To study the interaction between the target proteins in the treatment of RA by *O.* *vulgaris*, a PPI network was constructed using the String Version 11.0 platform. We set a score greater than 0.7 to indicate high confidence (Szklarczyk et al., 2017; Deng et al., 2019; Zhou et al., 2019), set the species as *Homo sapiens* (human), and hid the nodes that were disconnected from the network, leaving the rest of the parameters as default (Liu et al., 2019). The network analyzer function in the Cytoscape 3.7.1 software was used to study the PPI network topology attributes and draw the PPI network diagram.

#### Enrichment Analysis of the Target Using Genome Ontology and the Kyoto Encyclopedia of Genes and Genomes

The KEGG pathway and GO enrichment analyses of 198 targets of *O.* *vulgaris* in the Clusterprofiler software package were carried out using R Version 4.0.2, and the results were visualized.

### Molecular Docking

To further investigate the possibility of interaction between the four selected compounds (verbascoside, isoacteoside, melampyroside, and tricin) and the ten key targets (AKT1, MAPK1, EGFR, HRAS, APP, HSP90AA1, PIK3CA, SRC, PIK3R1, and AR), the structure of the small molecule compounds downloaded from PubChem (https://pubchem.ncbi.nlm.nih.gov/) was saved in SDF format. The Chem 3D software was used to convert the SDF file into a * Mol2 file, and the 3D structure of the target protein (PDB format) was downloaded from the PDB database (https://www.rcsb.org/) (Burley et al., 2017). The PyMol software was used to dehydrate the proteolytic enzyme and remove the active center of the original ligand. At the same time, hydrogenation, charge adding, and non-polar hydrogen merging were carried out using the AutoDock Tools 1.5.6 software. We used AutoDock Vina 1.1.2 for docking operations (Tanchuk et al., 2016). The receptor-ligand pairs were sorted and screened according to the binding free energy [Affinity (kcal/mol)]. Binding energy less than 0 indicated that the compound and protein could spontaneously bind and interact with each other. The lower the energy, the more stable the molecular conformation. Generally, a binding energy of ≤5.0 kcal/mol indicated a good binding effect. The PyMol 2.3.2 software was used for visualization.

### Anti-Inflammatory Assay

#### Effects of the Target Compounds of *O. vulgaris* on Cell Viability

Logarithmic phase RAW 264.7 cells distributed as single-cells seeded in 96-well plates at a density of 1 × 10^5^ cells/ml were placed in a 37°C 5% CO_2_ incubator for 24 h. After the cells adhered to the plate, different concentrations (6.25, 12.5, 25, 50, 100, and 200 μM) of the target compounds of *O.* *vulgaris* (isoacteoside, verbascoside, tricin, and melampyroside) were added to the culture. The normal control group (without the extract) was established at the same time. Five parallel wells were used for each group. After 48 h of culture, 10 μl of the freshly prepared 5 mg/ml MTT solution was added to each well and the culture was incubated at 37°C for 4 h in the dark. The supernatant was discarded, and 150 µl of DMSO was added to each well. After oscillation for 10 min, the absorbance (OD 570 nm) of each well was measured using a microplate analyzer.

#### Cell Viability Assay and the Protective Effects of Target Compounds of *O. vulgaris* on LPS-Treated RAW 264.7 Cells

We performed this analysis using the methods described in *Chemical Materials and Extraction and Processing*. The group demarcation based on treatment in this study was as follows: control group normal cell culture for 24 h without any treatment; inflammation model group with cells stimulated using 1 μg/ml LPS for 24 h; negative control group with cells treated using different target compounds (isoacteoside, verbascoside, tricin, and melampyroside) of *O.* *vulgaris* at 100 μM for 20 h; experimental group with cells stimulated using 1 μg/ml LPS for 4 h and different concentrations (6.25, 12.5, 25, 50, and 100 μM) of different target compounds (isoacteoside, verbascoside, tricin, and melampyroside) of *O.* *vulgaris* for 20 h. Three parallel wells were set in each group. The cell supernatant of each group was collected after culture, and NO release in the cell supernatant was detected according to the Griess test described in the NO kit. Then, the absorbance was measured at 550 nm (Gong et al., 2020).

#### Enzyme-Linked Immunosorbent Assay

Logarithmic phase RAW 264.7 cells made into single-cell suspension cells under the scratch seeded in a 6-well plate at a density of 5 × 10^5^ cells/ml were placed at 37°C in a 5% CO_2_ incubator for 24 h. The group demarcation based on treatment in this study was as follows: control group with normal culture without any treatment; inflammation model group with cells stimulated with 1 μg/ml LPS for 24 h; negative control group with cells treated with different target compounds (isoacteoside, verbascoside, tricin, and melampyroside) of *O.* *vulgaris* at 100 μM for 20 h; experimental group with cells stimulated using 1 μg/ml LPS for 4 h and different concentrations (25, 50, 100 μM) with different target compounds (isoacteoside, verbascoside, tricin, and melampyroside) of *O.* *vulgaris* for 20 h. Three parallel wells were set in each group. The supernatant of each group was collected, centrifuged for 5 min, and the supernatant was carefully removed for storage. The expression levels of TNF-*α*, IL-6, and IL-1*β* were detected using an ELISA kit.

### Statistical Analysis

All data were analyzed using SPSS 25.0, and the results were expressed as mean ± standard deviation (SD) of the three independent experiments. Differences were considered statistically significant if *p* < 0.05.

## Results

### Screening of the Protective Effects of *O. vulgaris* Extracts on LPS-Induced Inflammation in RAW 264.7 Cells

#### Determination of Indomethacin Concentration

**FIGURE 3:**
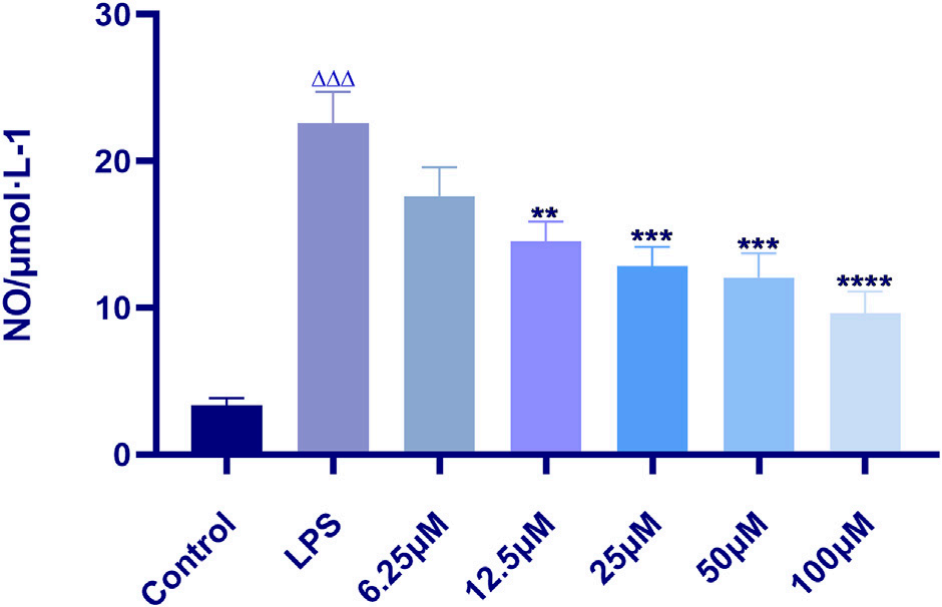
Effect of indomethacin on NO production in RAW 264.7 induced by LPS. Data are presented as means ± SD, *n* = 5. ** means ***p*** < 0.01, *** means ***p*** < 0.001, **** means ***p*** < 0.0001 compared with LPS group. ^△△△^ means ***p*** < 0.001 compared with control group.

The results showed that indomethacin could significantly inhibit the content of NO (*p* < 0.0001), and the inhibitory effect was proportional to the concentration of indomethacin (i.e., the higher the concentration, the stronger the inhibitory effect). The IC_50_ value of indomethacin was 58.82 μM in linear regression analysis. Therefore, 60 μM indomethacin was selected as the positive drug concentration for subsequent experiments (Figure 3).

#### Effects of the Crude Extracts of *O. vulgaris* on Cell Viability

**FIGURE 4:**
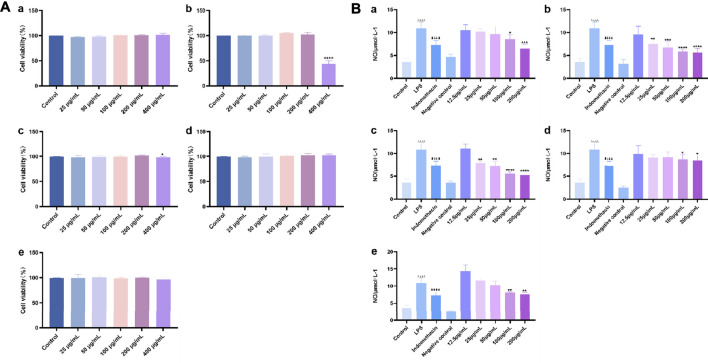
**(A)** Effect of the four *O.* *vulgaris* extracts on viability of RAW 264.7. **(Aa)** alcohol extract. **(Ab)** ethyl acetate extract. **(Ac)** n-butanol extract. **(Ad)** water extract. **(Ae)** petroleum ether extract. Data are presented as means ± SD, *n* = 5. * means ***p*** < 0.05, ** means ***p*** < 0.01, *** means ***p*** < 0.001, **** means ***p*** < 0.0001 compared with control group; **(B)** Effects of Different crude extracts of *O.* *vulgaris* on NO secretion in RAW 264.7 cells induced by LPS. **(Ba)** alcohol extract. **(Bb)** ethyl acetate extract. **(Bc)** n-butanol extract. **(Bd)** water extract. **(Be)** petroleum ether extract. Data are presented as means ± SD, *n* = 5. * means ***p*** < 0.05, ** means ***p*** < 0.01, *** means ***p*** < 0.001, **** means ***p*** < 0.0001 compared with LPS group. ^####^ means ***p*** < 0.0001 compared with LPS group. ^△△△△^ means ***p*** < 0.0001 compared with control group.

The effects of different polar fractions (alcohol, ethyl acetate, petroleum ether, water, and n-butanol) on the proliferation activity of mouse macrophage RAW 264.7 cells were detected. There were no significant differences in cell proliferation between the control group and the RAW 264.7 cells subjected to the different polar fractions of *O.* *vulgaris* (25, 50, 100, and 200 μg/ml) (Figure 4A; *p* > 0.05). When the concentration of the different polar fractions of *O.* *vulgaris* was greater than 200 μg/ml, the viability of the ethyl acetate- and n-butanol extract-treated cells decreased significantly (*p* < 0.01). The experimental results showed that the different polar fractions of *O.* *vulgaris* had no effect on cell proliferation and cytotoxicity at 25, 50, 100, and 200 μg/ml concentrations; thus, they were selected for subsequent experiments.

#### Cell Viability Assay and Protective Effects of the Extracts on LPS-Treated RAW 264.7 Cells

Analysis of the protective effects of the different crude extracts of *O.* *vulgaris* on LPS-induced inflammation in RAW 264.7 cells showed that the release of NO in the LPS model group was significantly higher than that in the control group (*p* < 0.0001). Compared with the LPS model group, the ethyl acetate (Figure 4B-b) and n-butanol fractions (Figure 4B-c) at concentrations of 25, 50, 100, and 200 μg/ml significantly inhibited the release of NO (*p* < 0.05) in a concentration-dependent manner. This indicated that the ethyl acetate and n-butanol fractions could improve the inflammatory response in LPS-induced mouse macrophage RAW 264.7 cells in the experimental concentration range. IC_50_ values were calculated according to the concentrations of the ethyl acetate and n-butanol fractions of *O.* *vulgaris*. The n-butanol fraction (135.9 μg/ml) showed a higher anti-inflammatory effect than the ethyl acetate fraction (166.8 μg/ml). The alcohol, water, and petroleum ether fractions (Figures 4B-a,d,e) had an inhibitory effect on NO secretion by RAW 264.7 cells stimulated with LPS at 100 and 200 μg/ml concentrations that showed a statistically significant difference to that on NO secretion in the LPS model group (*p* < 0.05). There was no statistically significant inhibitory effect at other concentrations (*p* > 0.05).

### Chemical Isolation of Pure Compounds

**FIGURE 5:**
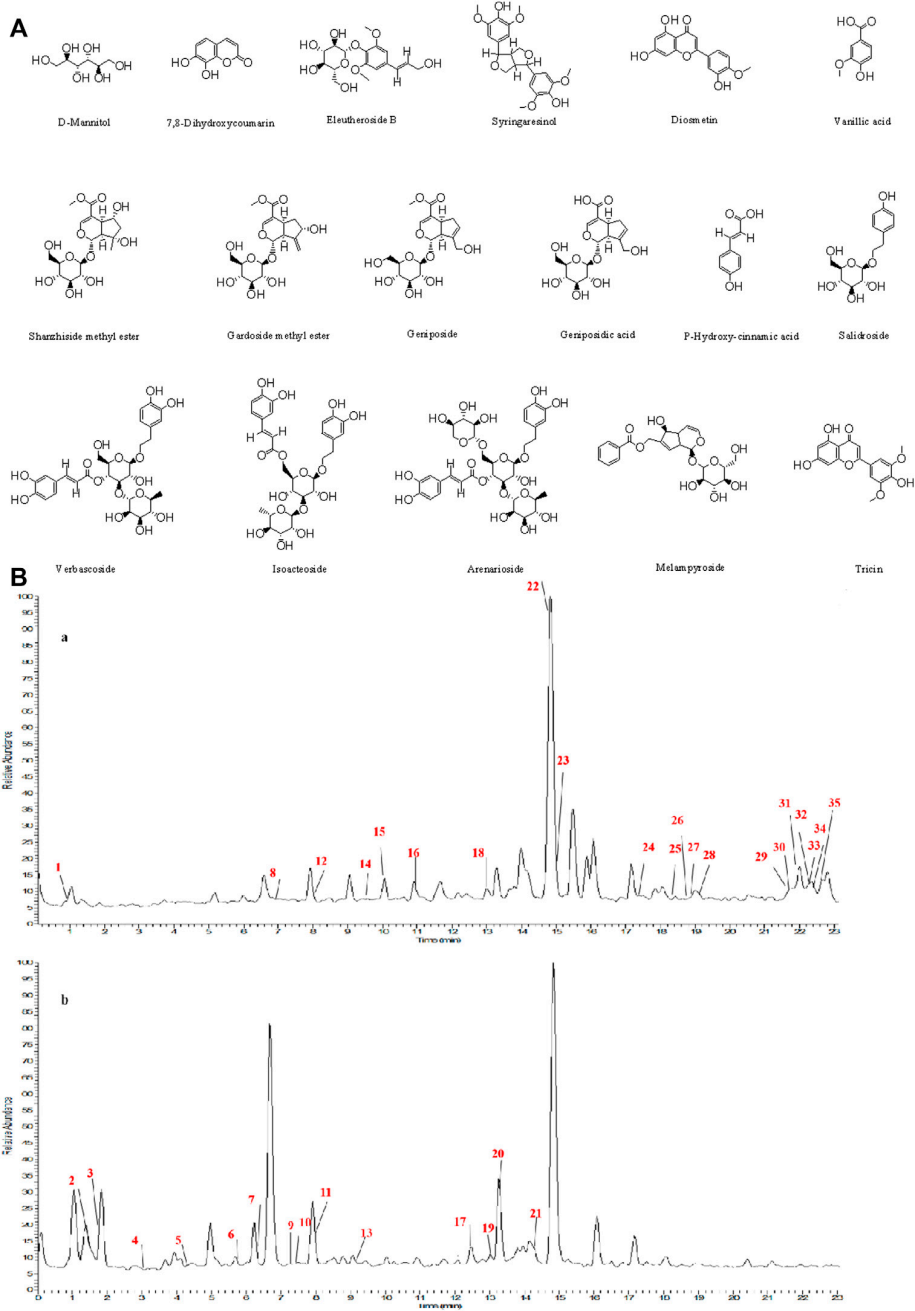
**(A)** The chemical structures of 17 monomeric compounds from *O.* *vulgaris*. **(B)** The total ion chromatograms (TIC) of ethyl acetate extract **(Ba)** and n-butanol extract **(Bb)** form the *O.* *vulgaris* in Positive mode.

The structures of the following 17 compounds isolated from *O.* *vulgaris* were identified by (^1^H-NMR, ^13^C-NMR and ESI-MS) techniques: D-mannitol (**compound 1**) (Wang 2015), geniposidic acid (**compound 2**) (Ge et al., 2017), salidroside (**compound 3**) (Qu et al., 2020), shanzhiside methyl ester (**compound 4**) (Zhang et al., 2019a), eleutheroside B (**compound 5**) (Meng et al., 2020), geniposide (**compound 6**) (Li et al., 2016), 7,8-dihydroxycoumarin (**compound 7**) (Zhou et al., 2021), gardoside methyl ester (**compound 8**) (Cai et al., 2013), arenarioside (**compound 9**) (Pan 2011), verbascoside (**compound 10**) (Zhang et al., 2019a), isoacteoside (**compound 11**) (Zhang et al., 2019a), vanillic acid (**compound 12**) (Wang et al., 2020c), p-hydroxy-cinnamic acid (**compound 13**) (Shang et al., 2019), melampyroside (**compound 14**) (Vogl et al., 2013), syringaresinol (**compound 15**) (Xia et al., 2021), tricin (**compound 16**) (Zhou et al., 2021), and diosmetin (**compound 17**) (Yang et al., 2020). The structures of the 17 monomers are shown in Figure 5A. Of them, compounds 1–9 and 12–17 were isolated from *O.* *vulgaris* for the first time.

### UPLC-MS Analysis

Using UPLC–MS, 35 compounds were identified from the n-butanol and ethyl acetate fractions, of which 16 were identified by the reference substance. The chemical compounds of the n-butanol and ethyl acetate fractions were preliminarily determined and were mainly divided into four categories: phenylethanoid glycosides (verbascoside, isoacteoside, arenarioside, salidroside), flavonoids (kaempferol, cynaroside, luteolin, luteolin-7-*O*-glucuronide, apigenin, apigenin-7-*O*-glucoside, tricin, chrysoeriol, quercetin, diosmetin, hydroxygenkwanin), iridoids (melampyroside, shanzhiside methyl ester, aucubin, geniposide, geniposidic acid, shanzhiside methyl ester), phenolic acids (caffeic acid, trans-cinnamic acid, 2-hydroxycinnamic acid, salicylic acid, ferulic acid, 7,8-dihydroxycoumarin), and others (adenosine, syringaresinol, D-Mannitol, esculetin). The total ion chromatograms of the ethyl acetate and n-butanol extracts of *O. vulgaris*, both in the positive ion mode, are presented in Figure 5B. The retention time, chemical formula, measured mass, and theoretical mass of the 35 identified compounds are presented in Table 1.

**TABLE 1:** Identification of major chemical compounds in n-butanol extract and ethyl acetate extract form the *O.* *vulgaris*.

Peak no	Extracted parts	RT [min]	Compounds	Chemical fomula	Ion mode	Measured value (*m/z*) (C/kg)	Theoretical value (*m/z*) (C/kg)	Error (ppm)	Fragmention	Reference
1	ethyl acetate extract	0.97	D-Mannitol^a^	C6H14O6	Negative	181.0710	181.0710	0.30404	101.0230,71.0124,69.0330	Zhang et al. (2019b)
2	n-butanol extract	1.40	Adenosine	C10H13N5O4	Negative	266.0895	266.0897	−0.90308	134.0460,107.0349,92.0240,80.4419	Zhang et al. (2019b)
3	n-butanol extract	1.79	Aucubin	C15H22O9	Negative	345.1187	345.1180	2.0421	165.0548,121.02823,71.0123,59.0124	Hu et al. (2015)
4	n-butanol extract	3.07	Geniposidic acid^a^	C16H22O10	Negative	373.1138	373.1129	2.4318	149.0595,123.0436,105.0332	Zhang et al. (2017)
5	n-butanol extract	4.39	Salidroside^a^	C14H20O7	Negative	299.1135	299.1125	3.3299	119.04917	Guo et al. (2014)
6	n-butanol extract	5.69	Shanzhiside methyl ester^a^	C17H26O11	Negative	405.1395	405.1391	0.0807	121.0649,101.0229,97.2474,68.9966	−
7	ethyl acetate extract	6.43	Esculetin	C9H6O4	Positive	179.0701	179.0702	−0.1719	161.0596,133.0648,123.0442	Yang et al. (2017)
8	ethyl acetate extract	6.96	Caffeic acid	C9H8O4	Negative	179.0342	179.0339	0.3097	135.0441,107.0350	He et al., 2014; Su et al., 2020
9	n-butanol extract	7.35	Salicylic acid	C7H6O3	Negative	137.0232	137.0233	−0.9282	93.0332	Xu et al. (2019)
10	n-butanol extract	7.39	Eleutheroside B^a^	C17H24O9	Positive	373.1487	373.1493	-0.6271	211.0962,193.0861,179.0701	Song and Feng, (2014)
11	n-butanol extract	7.97	Geniposide^a^	C17H24O10	Negative	387.1290	387.1286	2.4367	−	−
12	ethyl acetate extract	8.07	7,8-Dihydroxycoumarin^a^	C9H6O4	Positive	179.0701	179.0703	−0.1261	161.0234,133.0648,123.08081	−
13	n-butanol extract	9.06	Gardoside methyl ester	C17H24O10	Positive	389.1440	389.1442	−0.6988	227.0914, 195.0652, 177.0545, 149.0597	−
14	ethyl acetate extract	9.59	Vanillic acid^a^	C8H8O4	Negative	167.0340	167.0339	0.0809	152.0105,123.0074,108.0205	Luo et al. (2019)
15	ethyl acetate extract	9.99	p-Hydroxy-cinnamic acid^a^	C9H8O3	Negative	163.0390	163.0390	0.3764	147.0440,119.0493,91.0547	−
16	ethyl acetate extract	11.08	Ferulic acid	C10H10O4	Negative	193.0499	193.0496	1.8698	178.0263,149.0599,134.0361	Gui and Zheng, 2018; Luo et al., 2019
17	n-butanol extract	12.45	Arenarioside^a^	C34H44O19	Negative	755.2392	755.2393	−0.1437	161.0233,133.02821	−
18	ethyl acetate extract	13.01	Luteoloside	C21H20O11	Negative	447.0934	447.0922	2.7434	284.0331,227.0351,151.0025	Qiu et al. (2013)
19	n-butanol extract	13.07	Luteolin-7-O-glucuronide	C21H18O12	Negative	461.0725	461.0715	2.2273	285.0401	Shi et al. (2018)
20	n-butanol extract	13.35	Verbscoside^a^	C29H36O15	Negative	623.1974	623.1971	0.5473	313.8787,161.0232,133.0282	Wu et al. (2020)
21	n-butanol extract	14.22	Isoacteoside^a^	C29H36O15	Negative	623.1971	623.1971	0.1555	179.0338,161.0232,133.0282	Tong et al. (2019)
22	ethyl acetate extract	14.81	Melampyroside	C22H26O10	Negative	449.1458	449.1442	3.4713	213.1622,173.0118,121.0283	−
23	ethyl acetate extract	14.91	Apigenin-7-O-glucoside	C21H20O10	Negative	431.0978	431.0973	1.1718	268.0379,151,0022	Cvetanović et al. (2015)
24	ethyl acetate extract	17.31	Daidzein	C15H10O4	Positive	255.0649	255.0652	−1.1956	227.0697,199.0752,137.0235	−
25	ethyl acetate extract	18.37	Syringaresinol^a^	C22H26O8	Positive	419.1694	419.1700	−1.5295	330.1094,315.0864,181.0496,167.0702	−
26	ethyl acetate extract	18.81	Trans-cinnamic acid	C9H8O2	Positive	149.0232	149.0233	−0.5463	149.0233,130.9661,121.0286,103.0546	Zhang et al. (2013b)
27	ethyl acetate extract	18.86	Quercetin	C15H10O7	Negative	301.0355	301.0343	3.9276	151.0029,121.0282,107.0122,65.0017	Yang et al. (2016)
28	ethyl acetate extract	19.07	Luteolin	C15H10O6	Negative	285.0405	285.0394	4.0797	151.0026,133.0284,107.0125	Luo et al., 2019; Wang et al., 2018
29	ethyl acetate extract	21.88	Chrysoeriol	C16H12O6	Negative	299.0559	299.0550	3.0904	−	−
30	ethyl acetate extract	21.91	Apigenin	C15H10O5	Negative	269.0456	269.0445	4.1364	151.0026,117.0333,107.0124	Du et al. (2018)
31	ethyl acetate extract	22.13	Tricin^a^	C19H18O8	Negative	329.0667	329.0656	3.4408	314.0436,299.0196,271.0249,243.0293	Rui et al. (2011)
32	n-butanol extract	22.23	Kaempferol	C15H10O6	Negative	285.0407	285.0394	4.6150	169.5404,133.0285,107.7180,93.0334	Deng and Zhai (2018)
33	ethyl acetate extract	22.33	Hydroxygenkwanin	C16H12O6	Positive	301.0704	301.0707	−0.9667	286.0468,258.05212	Tao et al. (2018)
34	ethyl acetate extract	22.35	5,7,4′-Trihydroxy-6-methoxyisoflavone^a^	C16H12O6	Negative	299.0560	299.0550	3.1924	284.0326,256.0375,227.0350,151.0027	−
35	ethyl acetate extract	22.57	Diosmetin^a^	C16H12O6	Negative	299.0561	285.0394	3.9726	284.0322,256.0376,227.0348,151.0024	Shi et al. (2018)

a Compared with standard compounds.

The characteristics of the representative compounds in the ethyl acetate and n-butanol fractions of the *O.* *vulgaris* were as follows: given its molecular ion peak of *m/z* 449.1458 in negative ion mode, the molecular formula of peak 22 (melampyroside) was determined to be C_22_H_26_O_10_. Accordingly, a second-order spectrum was found, and fragment ion peaks at *m/z* 213.1622, 173.0118, and 121.0283 were recorded. Caffeic acid (peak 8) was detected in the ethyl acetate fraction of the herb, and its fragment ion peaks were at *m/z* 135.0441 and 107.0350. Peak 20 of n-butanol showed a molecular ion peak at *m/z* 623.1974, which was identified as that of verbascoside. There were fragment ions peaks at *m/z* 313.8787, 161.0232, and 133.0282 in the secondary spectrum, which was consistent with the fragmentation mode of the reference substance and that in previous reports. The molecular ion peak at *m/z* 623.1971 of peak 21, which was speculated to be that of the isomer of verbascoside, also showed a similar fragmentation mode. On comparison with the fragmentation mode of the reference substance, peak 21 was identified as that of isoacteoside. Peak 27 at *m/z* 301.0355 in the ethyl acetate fraction was identified as that of quercetin. The fragment ions with peaks at *m/z* 151.0029, 121.0282, 107.0122, and 65.0017 were found in the secondary spectrum, which was consistent with the fragmentation mode of the reference substance.

### Network Pharmacology

#### Chemical Composition of *O. vulgaris*

The known and predicted targets of the 36 compounds, after eliminating duplicates, were examined (Table 2). In this study, we only included results with probability >0.1 as the prediction target, and there were 294 targets in total.

**TABLE 2:** 36 compounds of *O.* *vulgaris*.

NO.	Name	Chemical formula	Pubchem-ID	Chemical formula
1	Kaempferol	C_15_H_10_O_6_	5280863	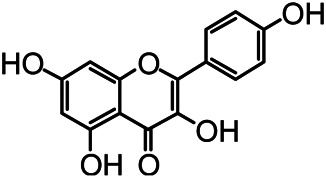
2	Quercetin	C_15_H_10_O_7_	5280343	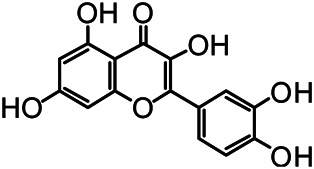
3	Apigenin	C_15_H_10_O_5_	5280443	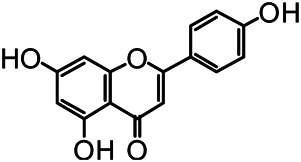
4	Luteolin	C_15_H_10_O_6_	5280445	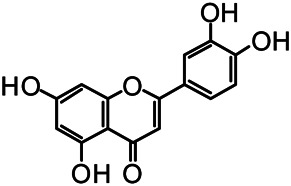
5	Cynaroside	C_21_H_20_O_11_	5280637	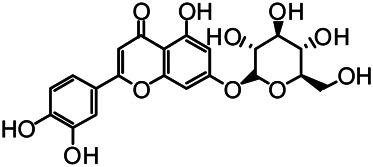
6	Chrysoeriol	C_16_H_12_O_6_	5280666	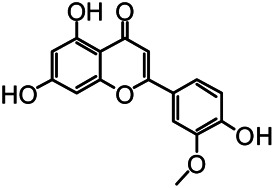
7	Caffeic acid	C_9_H_8_O_4_	689043	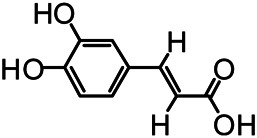
8	Isoacteoside	C_29_H_36_O_15_	6476333	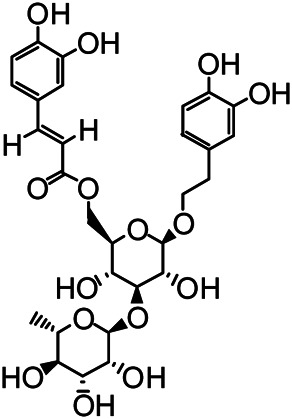
9	Verbascoside	C_29_H_36_O_15_	5281800	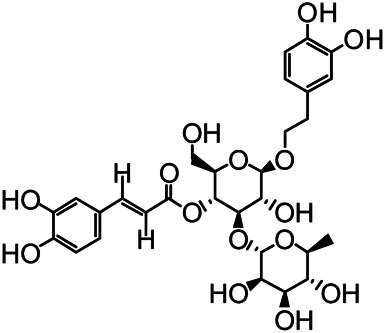
10	Rutin	C_27_H_30_O_16_	5280805	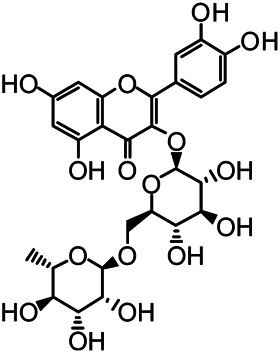
11	Arenarioside	C_34_H_44_O_19_	6442994	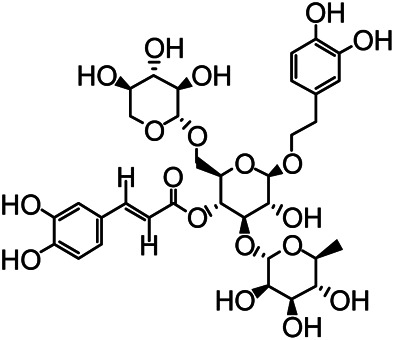
12	Tricin	C_17_H_14_O_7_	5281702	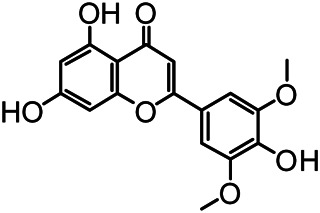
13	Apigenin-7-*O*-glucoside	C_21_H_20_O_10_	12304093	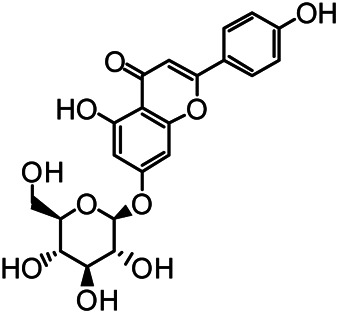
14	Adenosine	C_10_H_13_N_5_O_4_	60961	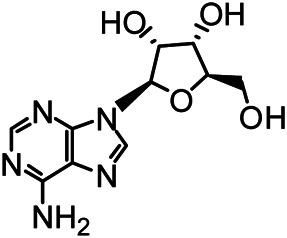
15	Daidzein	C_15_H_10_O_4_	5281708	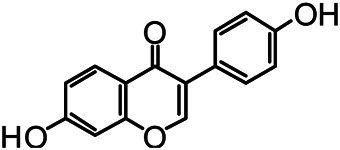
16	p-Hydroxy-cinnamic acid	C_9_H_8_O_3_	637542	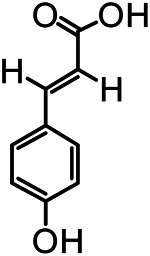
17	3, 7-di-*O*-methylquercetin	C_17_H_14_O_7_	5280417	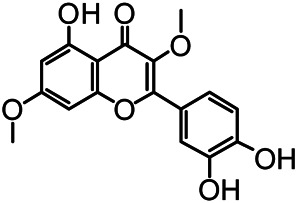
18	Hydroxygenkwanin	C_16_H_12_O_6_	5318214	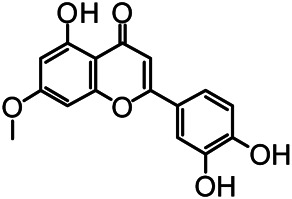
19	Luteolin-7-*O*-glucuronide	C_21_H_18_O_12_	13607752	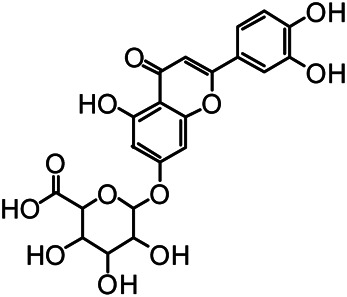
20	2-Hydroxycinnamic acid	C_9_H_8_O_3_	637540	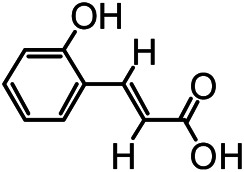
21	Ferulic Acid	C_10_H_10_O_4_	445858	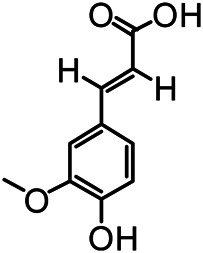
22	Salicylic acid	C_7_H_6_O_3_	338	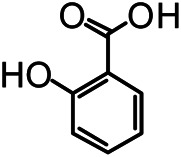
23	Diosmetin	C_16_H_12_O_6_	5281612	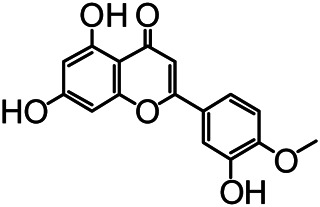
24	5,7,4′-Trihydroxy-6-methoxyisoflavone	C_16_H_12_O_6_	5281811	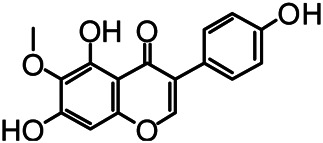
25	7,8-Dihydroxycoumarin	C_9_H_6_O_4_	5280569	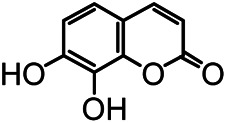
26	Esculetin	C_9_H_6_O_4_	5281416	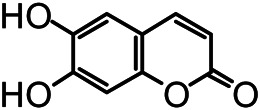
27	Verbascoside	C_15_H_22_O_9_	91458	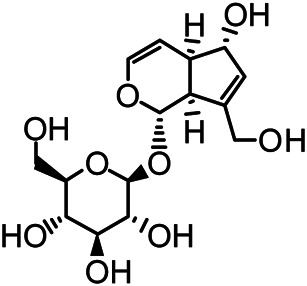
28	Trans-cinnamic acid	C_9_H_8_O_2_	444539	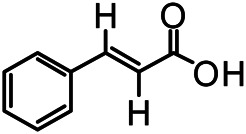
29	Gardoside methyl ester	C_17_H_24_O_10_	51692952	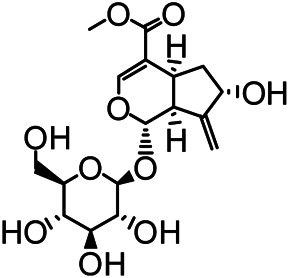
30	Shanzhiside methyl ester	C_17_H_26_O_11_	13892722	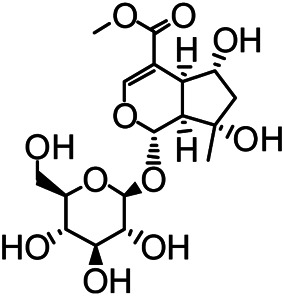
31	Geniposidic acid	C_16_H_22_O_10_	443354	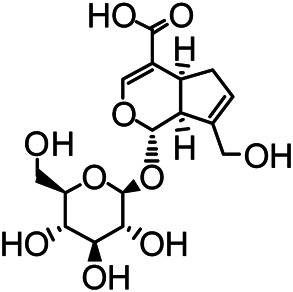
32	Melampyroside	C_22_H_26_O_10_	5319332	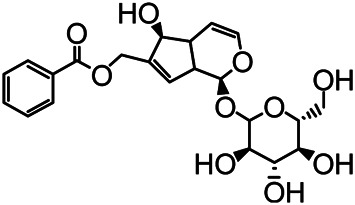
33	Syringaresinol	C_22_H_26_O_8_	100067	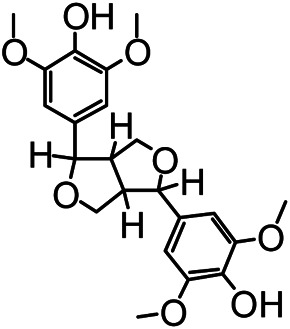
34	D-Mannitol	C_6_H_14_O_6_	6251	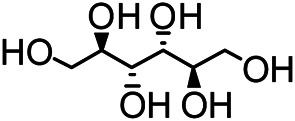
35	Eleutheroside B	C_17_H_24_O_9_	5316860	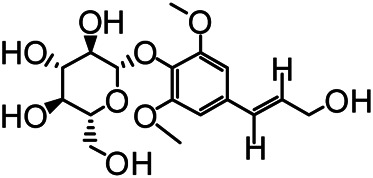
36	Vanillic acid	C_8_H_8_O_4_	8468	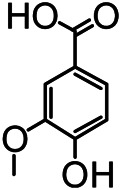

#### Targets Relevant to *O. vulgaris* Treatment of RA

**FIGURE 6:**
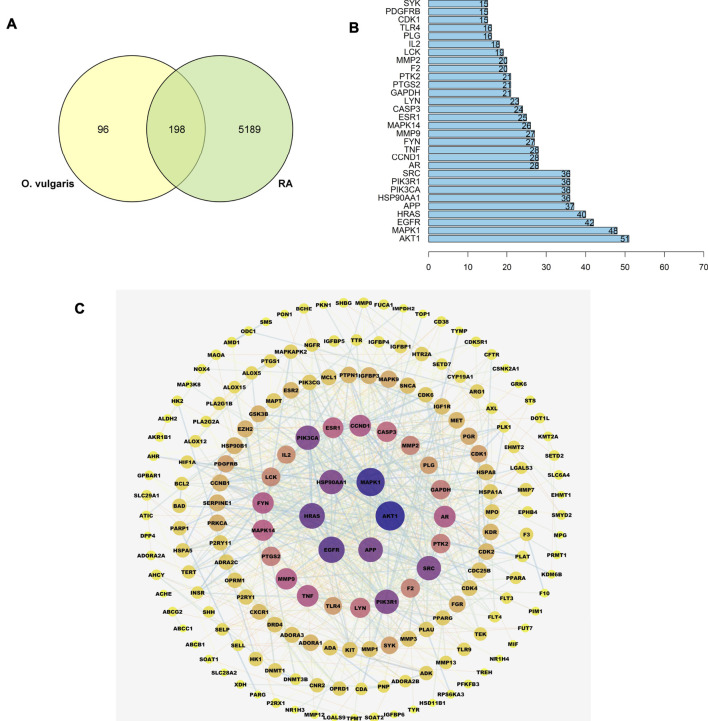
**(A)** Overlapping genes between RA-related genes and 36 compounds of *O. vulgaris* related genes; **(B)** The core gene for *O.* *vulgaris* treat RA; **(C)** PPI network diagram.

A total of 5,387 RA-related genes were compared with the target genes of 36 compounds to determine the potential therapeutic effect of each compound on RA target genes. The potential processing capacity of *O. vulgaris* to affect RA target genes was comprehensively obtained, and a total of 198 target genes were reported (Figure 6A).

#### Construction and Analysis of the PPI Network of the Target Protein

A PPI network of the candidate target protein of *O. vulgaris* was constructed. A visual PPI network diagram was generated to evaluate the topology parameters of the network nodes. A total of 198 nodes and 552 edges were obtained from these interactions. Nodes that were larger and darker in blue were those with higher degrees of freedom (Figure 6C). In PPI networks, nodes with a higher degree may play a more important role; thus, the top 30 proteins here may be key molecular targets for the treatment of RA. Further screening showed that 28 compounds were linked to the top 30 proteins, of which 14 were flavonoids, two were iridoids, three were phenylethanolglycosides, six were phenolic acids, and three were other compounds (Table 3). Akt1, MAPK1, EGFR, and HRAS were found to be associated with 51, 48, 42, and 40 proteins, respectively (Figure 6B; Table 4).

**TABLE 3:** The corresponding compounds of the 30 core genes.

NO.	Pubchem-ID	Compound	Target	Probability
P1	5,280,569	7,8-Dihydroxycoumarin	EGFR/PDGFRB/AKT1/LYN/SRC/PTK2	1/0.12/0.12/0.12/0.11/0.11
P2	60,961	Adenosine	GAPDH/EGFR/SRC/MAPK1/CCND1	0.24/0.13/0.12/0.11/0.11
P3	5,280,443	Apigenin	ESR1/CDK1/PTGS2/SYK/APP/MMP9/MMP2/AR/EGFR/CDK1/LCK/F2/PLG/PIK3R1/SRC/PTK2/AKT1	1/1/1/1/0.37/0.37/0.37/0.25/0.21/0.19/0.15/0.11/0.11/0.11/0.11/0.11/0.11
P4	12,304,093	Apigenin-7-*O*-glucoside	TNF/IL2/HSP90AA1	1/0.66/0.12
P5	689,043	Caffeic acid	MMP9/MMP2	0.18/0.18
P6	5,280,666	Chrysoeriol	CDK1/PLG/APP/SYK/MMP9/MMP2/ESR1/EGFR/PTGS2/F2/CDK1/PIK3R1/SRC/PTK2/AKT1/AR	0.30/0.25/0.24/0.21/0.21/0.21/0.14/0.14/0.13/0.12/0.12/0.12/0.12/0.12/0.12/0.12
P7	5,280,637	Cynaroside	TNF/IL2/PTGS2/HSP90AA1/PLG	1/1/0.11/0.11/0.11
P8	5,281,612	Diosmetin	PLG/CDK1/APP/SYK/MMP9/MMP2/ESR1/EGFR/F2/PTGS2/CDK1/PIK3R1/SRC/PTK2/AKT1	0.25/0.24/0.20/0.18/0.18/0.18/0.14/0.13/0.12/0.12/0.12/0.12/0.12/0.12/0.12
P9	5,281,416	Esculetin	EGFR	0.25
P10	51,692,952	Gardoside methyl ester	HSP90AA1/HRAS	0.11/0.11
P11	5,318,214	Hydroxygenkwanin	PLG/APP/MMP9/MMP2/SYK/SRC/ESR1/PTGS2/CDK1/PIK3R1/EGFR/AR/CDK1/LCK	1/0.24/0.16/0.16/0.14/0.13/0.12/0.12/0.12/0.12/0.12/0.11/0.11/0.11
P12	6,476,333	Isoacteoside	MMP2	0.65
P13	5,280,863	Kaempferol	SYK/MMP9/MMP2/CDK1/EGFR/F2/PIK3R1/SRC/PTK2/CDK1/AKT1/APP/ESR1/PTGS2	0.66/0.66/0.66/0.51/0.40/0.39/0.39/0.39/0.39/0.39/0.39/0.30/0.27/0.27
P14	5,280,445	Luteolin	CDK1/APP/SYK/MMP9/MMP2/ESR1/PTGS2/CDK1/EGFR/F2/PIK3R1/SRC/PTK2/AKT1/PLG/AR	1/1/1/1/1/0.39/0.37/0.30/0.27/0.27/0.27/0.27/0.27/0.27/0.15/0.14
P15	13607752	luteolin-7-*O*-glucuronide	TNF/IL2/PTGS2/EGFR/CASP3	0.13/0.13/0.1/0.1/0.1
P16	637,542	p-Hydroxy-cinnamic acid	MMP9/MMP2/TLR4/ESR1	0.22/0.22/0.13/0.12
P17	5,280,343	Quercetin	EGFR/F2/PIK3R1/SRC/PTK2/CDK1/MMP9/MMP2/AKT1/SYK/CDK1/APP	1/1/1/1/1/1/1/1/1/0.7/0.57/0.29
P18	100,067	syringaresinol	PIK3CA	0.11
P19	5,281,702	Tricin	PLG/APP/CDK1/MMP9/MMP2/SYK/ESR1/F2/EGFR/AR/CDK1/PIK3R1/SRC/PTK2/AKT1	0.19/0.15/0.11/0.11/0.11/0.11/0.10/0.10/0.10/0.10/0.10/0.10/0.10/0.10/0.10
P20	5,281,800	Verbascoside	MMP2	0.74/
P21	5,319,332	Melampyroside	HSP90AA1/EGFR/MAPK14	0.11/0.11/0.11
P22	637540	2-hydroxycinnamic acid	MMP9/MMP2/TLR4	0.13/0.13/0.13
P23	5,280,417	3,7-di-*O*-methylquercetin	PLG/APP/SRC/EGFR/PIK3R1/MMP9/MMP2/CDK1/SYK/F2/PTK2/PTGS2/CDK1/AKT1	1/0.23/0.15/0.12/0.11/0.11/0.11/0.11/0.10/0.10/0.10/0.10/0.10/0.10
P24	6,442,994	Arenarioside	MMP2	0.53
P25	5,316,860	Eleutheroside B	HRAS/EGFR	0.10/0.10
P26	8,468	Vanillic acid	FYN/LCK/MMP9/MMP2	0.13/0.13/0.13/0.13
P27	5,281,811	5,7,4′-Trihydroxy-6-methoxyisoflavone	EGFR/ESR1/IL2	1/0.15/0.14
P28	5,281,708	Daidzein	ESR1/EGFR/IL2	1/0.53/0.20

**TABLE 4:** The core gene for *O.* *vulgaris* treat RA.

Common name	Target	Uniprot ID	Degree	BetweennessCentrality
AKT1	Serine/threonine-protein kinase AKT	P31749	51	0.18667086
MAPK1	Mitogen-activated protein kinase 1	P28482	48	0.10010387
EGFR	Epidermal growth factor receptor erbB1	P00533	42	0.07068266
HRAS	Transforming protein p21/H-Ras-1	P01112	40	0.05861009
APP	Beta amyloid A4 protein	P05067	37	0.1461005
HSP90AA1	Heat shock protein HSP 90-alpha	P07900	36	0.05201668
PIK3CA	PI3-kinase p110-alpha subunit	P42336	36	0.0433,873
SRC	Tyrosine-protein kinase SRC	P12931	36	0.03284066
PIK3R1	PI3-kinase p85-alpha subunit	P27986	36	0.0433873
AR	Androgen Receptor	P10275	28	0.07071464
CCND1	cyclin D1	P24385	28	0.01586027
TNF	TNF-alpha	P01375	28	0.05159667
FYN	Tyrosine-protein kinase FYN	P06241	27	0.0156133
MMP9	Matrix metalloproteinase 9	P14780	27	0.07206925
MAPK14	MAP kinase p38 alpha	Q16539	26	0.01651102
ESR1	Estrogen receptor alpha	P03372	25	0.05512956
CASP3	Caspase-3	P42574	24	0.02237946
LYN	Tyrosine-protein kinase Lyn	P07948	23	0.01,350,012
GAPDH	Glyceraldehyde-3-phosphate dehydrogenase liver	P04406	21	0.02821895
PTGS2	Prostaglandin G/H synthase 2	P35354	21	0.05215256
PTK2	Focal adhesion kinase 1	PTK2	21	0.00276333
MMP2	Matrix metalloproteinase 2	P08253	20	0.01232746
F2	Thrombin	P00734	20	0.03351679
LCK	Tyrosine-protein kinase LCK	P06239	19	0.00821158
IL2	Interleukin-2	P60568	18	0.00452127
TLR4	Toll-like receptor 4	O00206	16	0.00642129
PLG	Plasminogen	P00747	16	0.01136883
PDGFRB	Platelet-derived growth factor receptor beta	P09619	15	0.00131725
CDK1	Cyclin-dependent kinase 1	P06493	15	0.00490717
SYK	Tyrosine-protein kinase SYK	P43405	15	0.00127815

#### GO Analysis of Target Proteins

**FIGURE 7:**
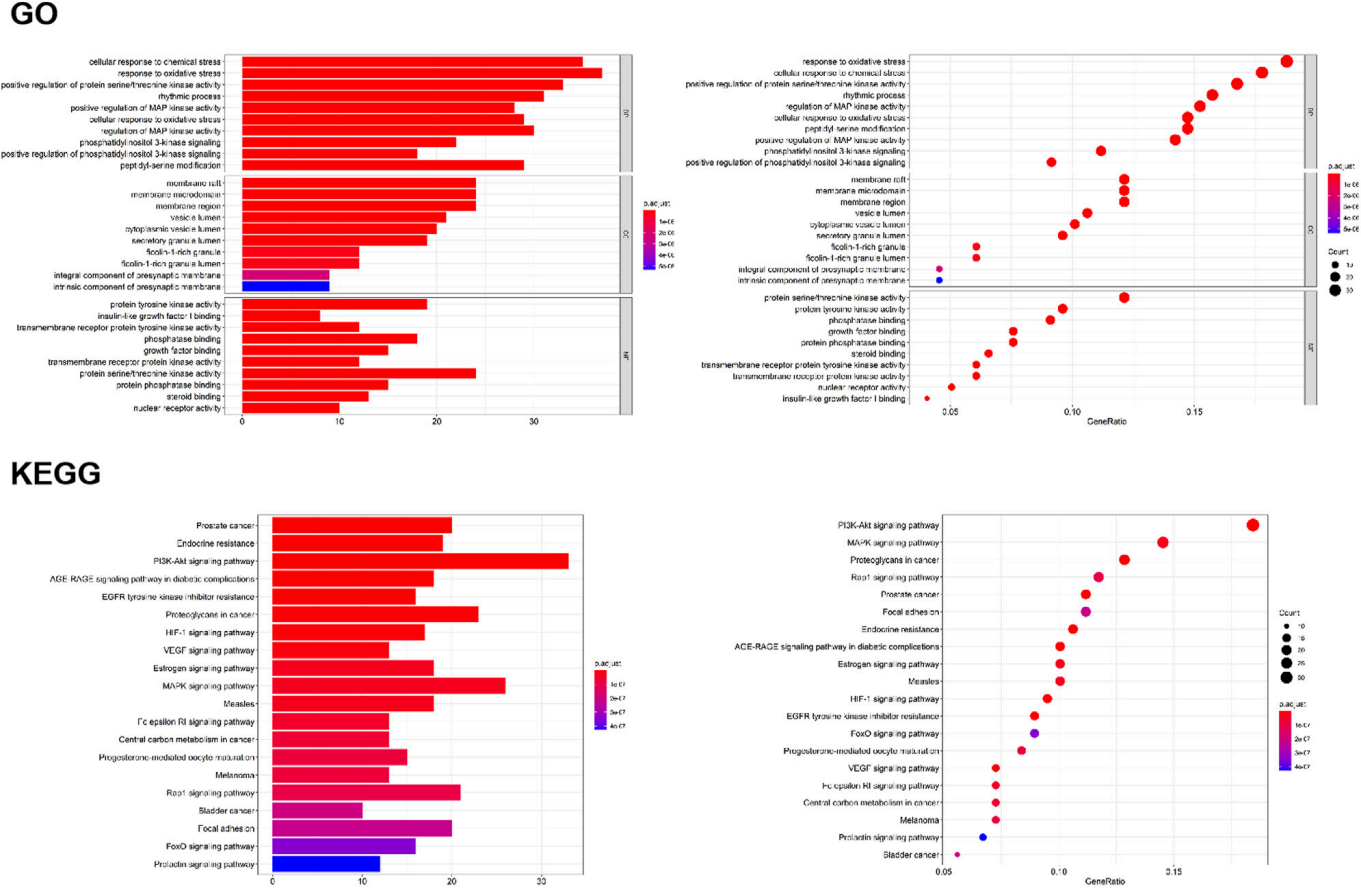
GO analyses of the 198 gene symbols associated with RA. [The x-axis showing the significant enrichment in the counts of these terms. The y-axis showing the categories of “biological process” in the GO of the target genes (*p* < 0.01)]; KEGG pathway enrichment analyses [The X-axis showing the counts of the target symbols in each pathway; the Y-axis showing the main pathways (*p* < 0.01)].

To verify the biological characteristics of 198 targets of *O. vulgaris*, GO enrichment analysis was performed on the presumed targets to clarify the related biological processes (Figure 7; *p* < 0.01). The top 10 significantly enriched terms in the BP (Biological Process), MF (Molecular Function), and CC (Cellular compound) categories were selected. Among them, BP (2,175 records), MF (167 records), and CC (92 records) accounted for 89.36, 6.86, and 3.78%, respectively. In the BP category, the target proteins were associated with cellular response to chemical stress (GO:0062197), response to oxidative stress (GO:0006979), and rhythmic processes (GO:0048511). In the MF category, the target proteins were associated with protein tyrosine kinase activity (GO:0004713), insulin-like growth factor I binding (GO:0031994), and phosphatase binding (GO:0019902). In the CC category, the target proteins were associated with membrane rafts (GO:0045121), membrane microdomains (GO:0098857), and vesicle lumen (GO:0031983).

#### KEGG Classification of Target Proteins

To determine the potential pathway underlying the effects of *O. vulgaris* on RA treatment, KEGG pathway enrichment analysis was performed on 198 genes. Figure 7 shows the 20 most significant signaling pathways. According to these results, the most important pathways associated with the effects of *O. vulgaris* on RA treatment were the PI3K-Akt, HIF-1, VEGF, estrogen, MAPK, Fc epsilon RI, Rap1, FoxO, and Prolactir signaling pathways, suggesting that these pathways mediated the effect of *O. vulgaris* on RA.

#### Construction of Compound-Target and Target-Pathway Network

**FIGURE 8:**
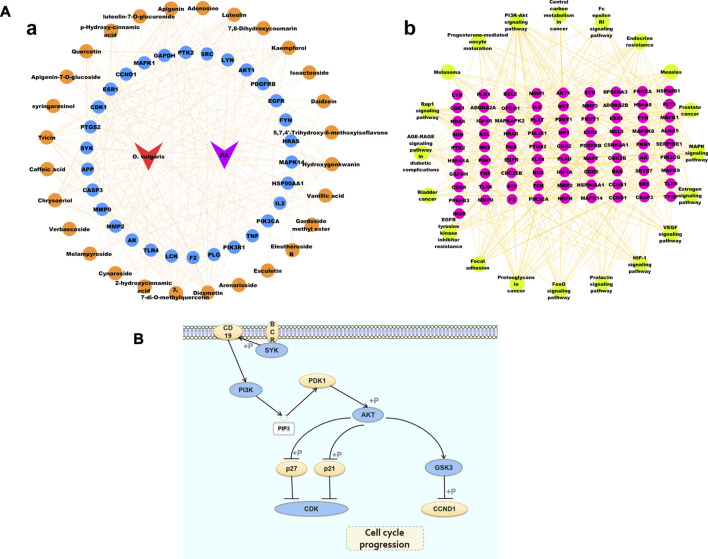
**(A)** Cytoscape Network diagram [**(Aa)**. Compound-Target diagram; **(Ab)**. Target-Pathway Diagram]; **(B)** PI3K-Akt signaling pathway.

Based on the target prediction results and KEGG analysis results of the chemical compounds of *O. vulgaris*, the Cytoscape 3.7.1 software was used to construct the network of the “compound-target” and “target and pathway” of *O. vulgaris* in the treatment of RA for visual analysis. In this network, the orange nodes represented compounds, blue and pink nodes represented targets, green nodes represented pathways, and red and purple nodes represented *O. vulgaris* and RA, respectively. The potential target of a compound was linked by an edge (Figure 8A). In this study, 30 core genes and their corresponding 36 compounds were used to establish an “compound-target” map, and 20 pathways from KEGG enrichment results and their corresponding targets were used to establish a “target-pathway” map.

Figure 8B shows the PI3K-Akt signaling pathway. The enrichment results showed that 17 common targets, namely, IGF1R, KDR, AKT1, PRKCA, PIK3R1, INSR, IL2, PTK2, CDK6, CDK2, GSK3B, FLT3, PIK3CG, SYK, KIT, MET, PKN1, and EGFR, were enriched in the PI3K-Akt signaling pathway.

### Compound Analysis

Verbascoside, isoacteoside, melampyroside, and tricin were isolated from the ethyl acetate and n-butanol fractions and were representative of the effective compounds. According to the area normalization method, the relative peak areas of verbascoside, isoacteoside, tricin, and melampyroside in the n-butanol and ethyl acetate fractions of *O. vulgaris* were up to 47.97% and 21.89%, respectively. Tricin is a flavonoid that presents anti-inflammatory activity. It acts mainly by inhibiting cyclooxygenase (COX), TNF and prostaglandin (PG), IL, NO, and other inflammatory mediators. It has potential medicinal value for the treatment of RA (Zhan et al., 2018; Yin et al., 2020). Verbascoside, and isoacteoside are phenylethanolglycosides that have been shown to have significant anti-inflammatory activity and potential value in the development of new drugs for RA (Song et al., 2011). Melampyroside is an iridoid compound, and its relative peak areas in the ethyl acetate and n-butanol extracts reached 11.39% and 13.31%, respectively. Iridoids have been studied for their anti-inflammatory activity (Gui and Zheng, 2018). Taken together, the four selected compounds were representative and formed a basis for the verification experiment.

### Molecular Docking

**FIGURE 9:**
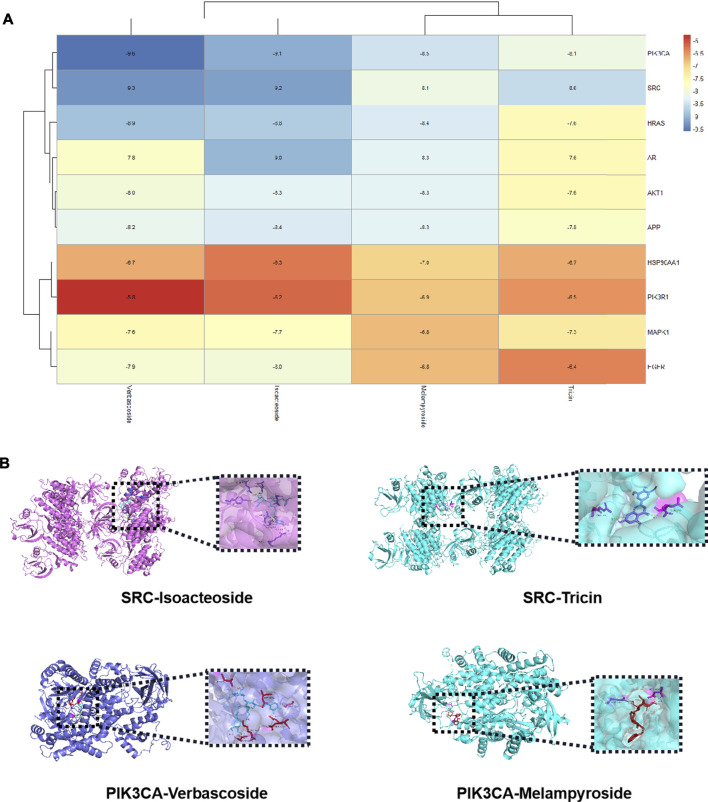
**(A)** Heat map of binding energy between the compounds of *O. vulgaris* and core targets molecule docking. **(B)** Docking modl diagram of compound and key target molecule.

We selected the top 10 targets (AKT1, MAPK1, EGFR, HRAS, APP, HSP90AA1, PIK3CA, SRC, PIK3R1, and AR) in the PPI network and four compounds (verbascoside, isoacteoside, melampyroside, and tricin) for analysis of molecular docking. The docking result is depicted as a heat map display in Figure 9A. The docking visualization results are shown in Figure 9B. The results show that the binding energy of each compound to protein was less than −5 kcal/mol, indicating that each compound could bind well to protein. Of them, the binding energies of isoacteoside and tricin to SRC were −9.2 and −8.6 kcal/mol, respectively. The binding energies of verbascoside and melampyroside were −9.6 and −8.5 kcal/mol, respectively.

### Anti-Inflammatory Assay

#### Protective Effects of Target Compounds on LPS-Treated RAW 264.7 Cells

**FIGURE 10:**
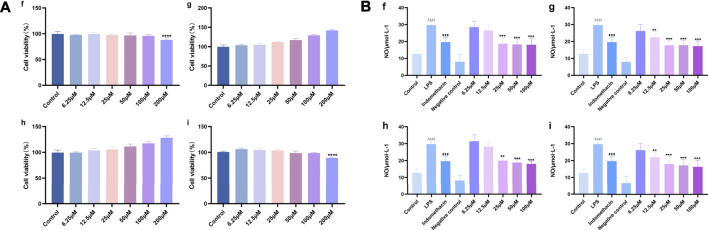
**(A)** Effect of target compounds of *O. vulgaris* extracts on viability of RAW 264.7 cell. **(Af)** melampyroside. **(Ag)** verbascoside. **(Ah)** isoacteoside. **(Ai)** tricin. Data are presented as means ± SD, *n* = 5. **** means ***p*** < 0.0001 compared with control group; **(B)** Effects of different target compounds of *O.* *vulgaris* on NO secretion in RAW 264.7 cells induced by LPS. **(Bf)** melampyroside. **(Bg)** verbascoside. **(Bh)** isoacteoside. **(Bi)** tricin. Data are presented as means ± SD, *n* = 5. * means ***p*** < 0.05, ** means ***p*** < 0.01, *** means ***p*** < 0.001, **** means ***p*** < 0.0001 compared with LPS group. ^####^ means ***p*** < 0.0001 compared with LPS group. ^△△△△^ means ***p*** < 0.0001 compared with control group.

The effects of different target compounds (melampyroside, isoacteoside, verbascoside, and tricin) on the proliferation activity of mouse macrophage RAW 264.7 cells were examined. The results showed that there was no significant difference between the proliferation of control cells and that of RAW 264.7 cells upon treatment with different target compounds of *O. vulgaris* at 6.25, 12.5, 25, 50, and 100 μM (*p* > 0.05). When the concentrations of melampyroside and tricin were more than 100 μM, cell viability decreased significantly (*p* < 0.0001). The different target compounds of *O. vulgaris* had no effect on cell proliferation and cytotoxicity at 25, 50, and 100 μM concentrations; therefore, the 25, 50, and 100 μM concentrations were selected for subsequent experiments (Figure 10A).

#### Cell Viability Assay and the Protective Effects of Target Compounds on LPS-Treated RAW 264.7 Cells

NO release in the LPS model group was significantly higher than that in the normal control group (Figure 9B; *p* < 0.0001). The effect of different target compounds on NO release was not significant in the negative control group, and there was no significant difference compared with that in the normal control group (*p* > 0.05). Compared with that in the LPS model group, treatment with 6.25, 12.5, 25, 50, and 100 μM of verbascoside (Figure 10B-g), and tricin (Figure 10B-i) significantly inhibited the release of NO (*p* < 0.05) in a concentration-dependent manner. Melampyroside (Figure 10B-f) and isoacteoside (Figure 10B-h) treatment significantly inhibited the release of NO at concentrations of 25, 50, and 100 μM (*p* < 0.05). The IC_50_ values were calculated according to the concentrations of the different target compounds of *O. vulgaris*. The IC_50_ values were as follows: melampyroside, 129.7 μM; verbascoside, 129.9 μM; isoacteoside, 114.5 μM; and tricin, 101.6. Thus, the anti-inflammatory effect of the four target compounds was in following order: tricin > isoacteoside > verbascoside > melampyroside.

#### Enzyme-Linked Immunosorbent Assay

**FIGURE 11:**
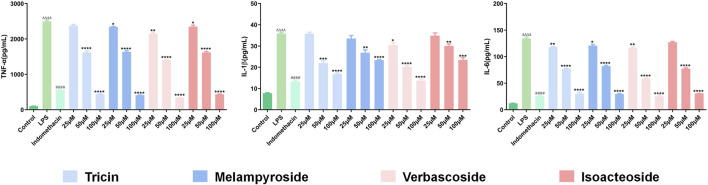
Effects of Different target compounds from *O. vulgaris* on TNF-*α*, IL-1*β* and IL-6 Content in RAW 264.7 Cells Induced by LPS. Data are presented as means ± SD, n = 5. * means ***p*** < 0.05, ** means ***p*** < 0.01, *** means ***p*** < 0.001, **** means ***p*** < 0.0001 compared with LPS group. ^####^ means ***p*** < 0.0001 compared with LPS group. ^△△△△^ means ***p*** < 0.0001 compared with control group.

Compared with those in the control group, the levels of TNF-*α*, IL-6, and IL-1*β* in the model group were significantly increased (Figure 11 **;** *p* < 0.0001), indicating that LPS caused an inflammatory response in RAW 264.7 cells. Compared with that in the model group, TNF-*α* content decreased significantly after treatment with different concentrations (25, 50, and 100 μM) of melampyroside, verbascoside, isoacteoside, and different concentrations (50 and 100 μM) of tricin. A concentration dependence was observed in the effective concentration analysis (*p* < 0.05). Compared with that in the model group, IL-1*β* content decreased significantly after treatment with different concentrations (25, 50, and 100 μM) of verbascoside, and different concentrations (50 and 100 μM) of tricin, isocalycoside, and melampyroside. A certain concentration dependence was observed (*p* < 0.05). Compared with that in the model group, IL-6 content decreased significantly after treatment with different concentrations (25, 50, and 100 μM) of melampyroside, verbascoside, and tricin, and different concentrations (50 and 100 μM) of isoacteoside. A concentration dependence was observed (*p* < 0.05). The results showed that melampyroside, verbascoside, isoacteoside, and tricin could downregulate the levels of inflammatory factors and exert anti-inflammatory effects. There was no significant difference seen between the verbascoside treated groups and the positive control group (indomethacin) in IL-1*β* content at 100 μM (*p* > 0.05). There was no significant difference in IL-6 content between the verbascoside, and tricin groups and the positive control group (indomethacin) at 100 μM (*p* > 0.05).

## Discussion

Based on the symptoms of RA and the pharmacodynamic basis of *O. vulgaris*, although *O. vulgaris* has enormous therapeutic potential, its full chemical characterization remains incomplete. Therefore, the purpose of this study was to evaluate the biological activity of *O. vulgaris* and analyze its chemical compounds to lay a foundation for the identification of new drugs.

In this study, anti-inflammatory activity was determined in terms of the NO content of RAW 264.7 mouse macrophages induced by LPS. The results showed that the ethyl acetate and n-butanol extracts exhibited better activity. We isolated 17 compounds from the n-butanol and ethyl acetate extracts of *O. vulgaris* with compounds 1–9 and 12–17 being isolated from *O. vulgaris* for the first time. The UPLC–Q-Exactive assay was used to identify 35 compounds from the ethyl acetate and n-butanol extracts of *O. vulgaris*.

Network pharmacological results showed that 36 compounds, including flavonoids, iridoids, phenolic acids, and phenylethanolglycosides, were directly related to the therapeutic effect of *O. vulgaris* on RA. In this study, the PI3K-Akt pathway was found to be particularly important for the treatment of RA by *O. vulgaris*. The PI3K-Akt signaling pathway is closely related to the occurrence and development of RA and plays an important role in the regulation of differentiation and formation of osteoblasts (Wang et al., 2019). Overproliferation of synovial cells and RA can be reversed via the use of P13K-Akt signaling inhibitors, or by the upregulation of endogenous negative regulatory proteins in the signaling pathway. This inhibits glycogen synthase kinase 3 (GSK3) activity and accelerates cell apoptosis. In RA, the earliest phenomenon is the formation of new blood vessels, which can develop into a pannus in severe cases. Vascular endothelial growth factors (VEGFs) are an important class of signaling proteins and ligands secreted by cells to stimulate vasculogenesis and angiogenesis (Da and Li, 2012). They are also as important inducers and direct pro-inflammatory factors. VEGF proteins play a vital role in activating angiogenesis in RA through the upregulation of the VEGF pathway (Yi et al., 2016; Meloa et al., 2020). Cheng et al. (2011) used the immunohistochemical SP method to detect VEGF protein expression in the synovial tissues of the knee joints of 27 patients with active RA. The results showed that the expression of VEGF in the synovial tissues of controls and patients with osteoarthritis was significantly lower than that in patients with RA.

According to the component-target interaction analysis, ten targets with higher degrees on the PPI network (AKT1, MAPK1, EGFR, HRAS, APP, HSP90AA1, PIK3CA, SRC, PIK3R1, and AR) were identified. The docking scores of all compound-target pairs were less than −5 kcal/mol, indicating that each of the four selected compounds (verbascoside, isoacteoside, melampyroside, and tricin) had good binding affinity to all 10 targets. The binding energies of isoacteoside, tricin to SRC were −9.2, −9.9, and −8.6 kcal/mol, respectively. The binding energies of verbascoside and melampyroside were −9.6 and −8.5 kcal/mol, respectively. The molecular docking simulation strengthened all the above investigations to some extent.

As a major pro-inflammatory factor in the pathogenesis of RA, TNF-*α* is considered the leading trigger for inflammation and joint destruction. It can stimulate the synovium, fibroblasts, osteoclasts, and other cells to produce destructive substances called matrix metalloproteinases, which can damage cartilage and gradually destroy bone tissue in patients with RA. Therefore, TNF-*α* level can be used as an indicator of inflammatory activity in RA (Wang et al., 2016). IL-6 is produced by synovial cells, and its abnormal expression and dysregulation are typical characteristics of RA [39]. IL-6 can enhance the effects of IL-1 and TNF-*α* and is considered to be an amplifying factor of some biological effects of TNF-*α* (Shi et al., 2013). At non-toxic doses, the four target compounds in *O. vulgaris* significantly reduced the secretion of TNF-*α*, IL-1*β*, IL-6, and NO, suggesting that *O. vulgaris* is a potential therapeutic agent for RA. The anti-inflammatory activity of *O. vulgaris* can be attributed to its bioactive compounds. Phenylethanolglycosides, flavonoids, and iridoids have a wide range of pharmacological activities, including anti-inflammatory activityies (Hanfer et al., 2017; Spagnuolo et al., 2018; Zhang et al., 2018). Verbascoside has shown anti-inflammatory activities, such as reducing the presence of pro-inflammatory factors in intestinal inflammation (Danae et al., 2013). As an isomeride of verbascoside, the similarities and differences in the anti-inflammatory activity of isoacteoside and the underlying mechanism of action require further exploration. Melampyroside is an iridoid terpene compound, and the relative peak area of the ethyl acetate and n-butanol fractions of *O. vulgaris* is relatively large. Therefore, pharmacological studies on *O. vulgaris* cannot be ignored. Tricin is a flavonoid, and its anti-inflammatory properties have been studied (Zhan et al., 2018; Yin et al., 2020); it is noteworthy that tricin has shown outstanding anti-inflammatory activity in *in vitro* experiments.

Based on studies of previous chemical substances, we used bioinformatics to predict the targets and potential action pathways of *O. vulgaris* in the treatment of RA and detected its target compounds from the levels of inflammatory factors *in vitro*. The results of network analysis and prediction showed that the PI3K-Akt pathway may play an important role in *O. vulgaris*-mediated treatment of RA. Therefore, the development of anti-inflammatory drugs using *O. vulgaris* has considerable advantages. However, bioinformatics methods still have some limitations, for example, it is found that it is too one-sided to explore the efficacy of medicinal plants only from the perspective of network pharmacology. It is necessary to consider the relative content of compounds in medicinal plants, so as to give better play to the pharmacological role of medicinal plants. In addition, the binding energy of compounds in molecular docking experiments is not necessarily at the same level as their therapeutic potential *in vivo*, and further systematic molecular biology experiments are needed to verify the accurate mechanisms.

In summary, the results of this study support the potential anti-inflammatory activity of *O. vulgaris*, and combined with computer pharmacology, it is speculated that it may have the activity of treating RA. In the development of Chinese and Mongolian medicine, *O. vulgaris* could be a potential source of novel drugs for the treatment of RA. Our study also provides a novel paradigm to identify the potential mechanisms of pharmacological effects derived from a natural compound.

## Data Availability

The original contributions presented in the study are included in the article/Supplementary Material, further inquiries can be directed to the corresponding authors.

## References

[B1] Bai P. Xin S. S. Dong Y. (2015). The Study Progress of Mongolian Medicine Treatment of Rheumatoid Arthritis. J. Inner Mongolia Med. Univ. (s1), 12–15. 10.16343/j.cnki.issn.2095-512x.2015.s1.003

[B2] Burley S. K. Berman H. M. Kleywegt G. J. Markley J. L. Nakamura H. Velankar S. (2017). Protein Data Bank (PDB): The Single Global Macromolecular Structure Archive. Methods Mol. Biol. 1607, 627–641. 10.1007/978-1-4939-7000-1_26 28573592 PMC5823500

[B3] Cai C. J. Zhang Z. L. Zuo Y. M. Zhu Y. Y. Luo G. M. Zhang J. (2013). Studies on the Chemical Components of Iridoids of *Gardenia Jasminoides* Ellis. Lishizhen Med. Mater. Med. Res. 24, 342–343. 10.3969/j.issn.1008-0805.2013.02.041

[B4] Carrillo-Ocampo D. Bazaldúa-Gómez S. Bonilla-Barbosa J. B. B. Aburto-Amar R. Rodríguez-López V. (2013). Anti-inflammatory Activity of Iridoids and Verbascoside Isolated from Castilleja Tenuiflora. Molecules 18, 12109–12118. 10.3390/molecules181012109 24084016 PMC6270386

[B5] Cheng Q. Song W. G. Wang N. Zhang Y. X. Zhang Y. P. (2011). Expression and Clinical Significance of VEGF in Rheumatoid Arthritis Synovium. Chin. Mod. Med. 18, 70–71. 10.3969/j.issn.1674-4721.2011.25.038

[B6] Cicek M. Unsal V. Doganer A. Demir M. (2021). Investigation of Oxidant/antioxidant and Anti-inflammatory Effects of Apigenin on Apoptosis in Sepsis-Induced Rat Lung. J. Biochem. Mol. Tox. e22743. 10.1002/jbt.22743 33605009

[B7] Cvetanović A. Švarc-Gajić J. Zeković Z. Savić S. Vulić J. Mašković P. (2015). Comparative Analysis of Antioxidant, Antimicrobiological and Cytotoxic Activities of Native and Fermented Chamomile Ligulate Flower Extracts. Planta 242, 721–732. 10.1007/s00425-015-2308-2 25976264

[B8] Da G. L. Li H. B. (2012). Vascular Endothelial Growth Factor Role in the Angiogenesis of Rheumatoid Arthritis and the Related Research Progress. Med. Recapit. 18, 644–648. 10.3969/j.issn.1006-2084.2012.05.002

[B9] Daina A. Michielin O. Zoete V. (2019). SwissTargetPrediction: Updated Data and New Features for Efficient Prediction of Protein Targets of Small Molecules. Nucleic Acids Res. 47, W357. 10.1093/nar/gkz382 31106366 PMC6602486

[B10] Deng J. L. Xu Y. H. Wang G. (2019). Identification of Potential Crucial Genes and Key Pathways in Breast Cancer Using Bioinformatic Analysis. Front. Genet. 10, 695. 10.3389/fgene.2019.00695 31428132 PMC6688090

[B11] Deng Y. F. Zhai X. J. (2018). Simultaneous Determination of Astragulin and Kaempferol in Bairui Granule by HPLC-MS/MS. China Pharm. 21, 524–526.

[B12] Du G. Fu L. Jia J. Pang X. Yu H. Zhang Y. (2018). Validated UPLC-MS/MS Method for Quantification of Seven Compounds in Rat Plasma and Tissues: Application to Pharmacokinetic and Tissue Distribution Studies in Rats after Oral Administration of Extract of Eclipta Prostrata L. Biomed. Chromatogr. 32, e4191. 10.1002/bmc.4191 29349861

[B13] Ferreira de Melo I. M. Martins Ferreira C. G. Lima da Silva Souza E. H. Almeida L. L. Bezerra de Sá F. Cavalcanti Lapa Neto C. J. (2020). Melatonin Regulates the Expression of Inflammatory Cytokines, VEGF and Apoptosis in Diabetic Retinopathy in Rats. Chemico-Biological Interactions 327, 109183. 10.1016/j.cbi.2020.109183 32554039

[B14] Ge W. Li H. B. Yu Y. Fang H. Meng Z. Q. Huang W. Z. (2017). Research on Chemical Constituents from Reduning Injection (IV). Chin. Tradit. Herb. Drugs 48, 3042–3050. 10.7501/j.issn.0253-2670.2017.15.005

[B15] Gong X. Wang J. Zhang M. Wang P. Wang C. Shi R. (2020). Bioactivity, Compounds Isolated, Chemical Qualitative, and Quantitative Analysis of *Cymbaria Daurica* Extracts. Front. Pharmacol. 11, 48. 10.3389/fphar.2020.00048 32116723 PMC7019114

[B16] Gui Q. Zheng J. (2018). Simultaneous Determination of Eight Components in *Angelica Sinensis* Based on UHPLC-ESI-MS/MS Method for Quality Evaluation. Biomed. Chromatogr. 33, e4326. 10.1002/bmc.4326 29956832

[B17] Guo N. Ding W. Wang Y. Hu Z. Wang Z. Wang Y. (2014). An LC-MS/MS Method for the Determination of Salidroside and its Metabolite P-Tyrosol in Rat Liver Tissues. Pharm. Biol. 52, 637–645. 10.3109/13880209.2013.863946 24479765

[B18] Guo X. Ji J. Feng Z. Hou X. Luo Y. Mei Z. (2020). A Network Pharmacology Approach to Explore the Potential Targets Underlying the Effect of Sinomenine on Rheumatoid Arthritis. Int. Immunopharmacol 80, 106201. 10.1016/j.intimp.2020.106201 31972421

[B19] Hanfer M. Cheriet T. Ameddah S. Mancini I. Seghiri R. Menad A. (2017). Iridoids and Anti-inflammatory Properties of N-Butanol Extract of Linaria Tingitana Boiss. & Reut. Nat. Prod. Res. 31, 2008–2015. 10.1080/14786419.2016.1272111 28076999

[B20] Hao M. Cheng T. Wang Y. Bryant H. S. (2013). Web Search and Data Mining of Natural Products and Their Bioactivities in PubChem. Sci. China Chem. 56, 1424–1435. 10.1007/s11426-013-4910-0 PMC3869387 24363665

[B21] He F. Zhang J. Mou J. L. Zheng L. Lan Y. Y. Huang Y. (2014). Simultaneous Determination and Pharmacokinetics of Albiflorin, Caffeic Acid and Chlorogenic Acid in Rat Plasma by UPLC-MS. Lishizhen Med. Mater. Med. Res. 25, 1803–1806.

[B22] Hu F. An J. Li W. Zhang Z. Chen W. Wang C. (2015). UPLC-MS/MS Determination and Gender-Related Pharmacokinetic Study of Five Active Ingredients in Rat Plasma after Oral Administration of *Eucommia Cortex* Extract. J. Ethnopharmacol. 169, 145–155. 10.1016/j.jep.2015.04.007 25910535

[B23] Jiang Y. Zhong M. Long F. Yang R. Zhang Y. Liu T. (2019). Network Pharmacology-Based Prediction of Active Ingredients and Mechanisms of *Lamiophlomis Rotata* (Benth.) Kudo against Rheumatoid Arthritis. Front. Pharmacol. 10, 1435. 10.3389/fphar.2019.01435 31849678 PMC6902022

[B24] Li J. B. Zhou Q. Chen F. F. Hua A. L. Wang S. Z. Wu M. T. (2019a). Pharmacokinetics and Bioavailability of Kaempferol in Rat Plasma by UPLC-MS/MS(Article). Lat. Am. J. Pharm. 38, 80–84.

[B25] Li L. Gu W. Y. (2020). Resveratrol-mediated Tenascin-c/TLR4 Signaling Pathway Regulating Inflammatory Reaction in RA Rats. West. J. Tradit. Chin. Med. 33, 20–23.

[B26] Li T. Gao D. Du M. Cheng X. Mao X. (2018). Casein Glycomacropeptide Hydrolysates Inhibit PGE2 Production and COX2 Expression in LPS-Stimulated RAW 264.7 Macrophage Cells via Akt Mediated NF-Κb and MAPK Pathways. Food Funct. 9, 2524–2532. 10.1039/c7fo01989k 29666854

[B27] Li X. He H. H. Lin D. Xie Y. Q. Xiao M. (2019b). Prediction of the Mechanism of Action of Paederia Scandens in the Treatment of Rheumatoid Arthritis Based on Network. Hainan Med. J. 30, 3133–3139. 10.3969/j.issn.1003-6350.2019.24.002

[B28] Li Y. Wang X. L. Song J. L. Qi H. Y. (2016). Chemical Constituents from Leaves of Gardeniae Fructus. Chin. J. Exp. Tradit. Med. Formulae. 22, 68–70.

[B29] Liu F. Liu Y. Zhan S. P. Lv J. Yu M. J. Xia P. Y. (2019). Mechanism of Tibetan Medicine Terminalia Chebula Retz in Rheumatoid Arthritis: a Study Based on Network Pharmacology. J. Third Mil. Med. Univ. 41, 2238–2245.

[B30] Liu Z. D. Jiang M. Tan Y. He X. J. Jia D. M. Zhou K. (2015). Research on Network Pharmacology of Cold or Heat Herbal Formula Correspond with Cold or Heat Syndromes of Rheumatoid Arthritis. Chin. J. Tradit. Chin. Med. Pharm. 30, 3191–3195.

[B31] Luo X. M. Su M. F. Chang X. Y. Wang X. M. Li Z. Z. Wang W. H. (2019). Qualitative and Quantitative Analysis of Main Chemical Constituents in Eucommia Ulmoides by LC-MS. Mod. Chin. Med. 21, 1029–1040. 10.13313/j.issn.1673-4890.20190129004

[B32] Mao G. H. Zhang L. Q. Qian F. Chen K. X. Xu J. W. Li J. M. (2019). Research Progress on Anti-inflammatory Activity of Three Kinds of Iridoid Glycosides in Chinese Materia Medica. Chin. Tradit. Herb. Drugs 50, 228–236.

[B33] Meng X. H. Shang X. Y. Yang J. L. (2020). Phenolic Chemical Constituents of *Zanthoxylum Schinifolium* Pericarps and Their Anti-oxidative Effects. Chin. Tradit. Herb. Drugs 51, 2095–2101. 10.7501/j.issn.0253-2670.2020.08.015

[B34] Nigro O. Tuzi A. Tartaro T. Giaquinto A. Vallini I. Pinotti G. (2020). Biological Effects of Verbascoside and its Anti-inflammatory Activity on Oral Mucositis: a Review of the Literature. Anticancer Drugs 31, 1–5. 10.1097/CAD.0000000000000818 31609769

[B35] Pan Y. N. (2011). Study on Chemical Constituents and Biological Activities of Fresh Products of *Cistanche Tubulosa*. Shenyang: Shenyang Pharmaceutical University.

[B36] Qiu F. Li Z. He L. Wang D. (2013). HPLC-ESI-MS/MS Analysis and Pharmacokinetics of Luteoloside, a Potential Anticarcinogenic Component Isolated from *Lonicera japonica*, in Beagle Dogs. Biomed. Chromatogr. 27, 311–317. 10.1002/bmc.2793 22865633

[B37] Qu S. Y. Wei X. D. Sai M. Lan X. Z. Chen M. (2020). Chemical Constituents from *Rhodiola Crenulata* . Chin. Tradit. Pat. Med. 42, 3199–3203.

[B38] Rui W. Feng Y. F. Liu S. J. Tan Y. Z. (2011). Analysis of Flavonoids in Ranunculus Japonicus by UPLC I-IILIC/Q-TOF-MS. Asia-pacific Tradit. Med. 7, 18–21.

[B39] Shang Z. M. Cheng L. Liu G. Y. Zhang M. S. Li X. F. Xiao S. J. (2019). Chemical Constituents of *Dendrobium Bellatulum* . Chin. Tradit. Herb. Drugs 50, 2036–2040. 10.7501/j.issn.0253-2670.2019.09.005

[B41] Shi F. Pan H. Lu Y. Ding L. (2018). An HPLC-MS/MS Method for the Simultaneous Determination of Luteolin and its Major Metabolites in Rat Plasma and its Application to a Pharmacokinetic Study. J. Sep. Sci. 41, 3830–3839. 10.1002/jssc.201800585 30101558

[B42] Shi L. P. He Y. Xun W. Wei Y. L. (2013). The Role of the Combination Therapy on the Levels of Pro-inflammatory Cytokines and Acute Phase Reactants in Rheumatoid Arthritis. Pract. J. Clin. Med. 10, 78–80.

[B43] Song G. X. Ma L. Y. Wei F. Ma S. C. (2011). The Distribution of Mangiferin Benzyl Ethanol Research Progress and Pharmacological Activities. Asia-pacific Tradit. Med. 7, 169–171.

[B44] Song Y. Feng X. S. (2014). UPLC-MS/MS Analysis of the Constituents in the Roots of A. Sessiliflorus and Comparison with A. Gracilistylus and A. Senticosus. Chin. J. Pharm. Anal. 34, 958–965.

[B45] Spagnuolo C. Moccia S. Russo G. L. (2018). Anti-inflammatory Effects of Flavonoids in Neurodegenerative Disorders. Eur. J. Med. Chem. 153, 105–115. 10.1016/j.ejmech.2017.09.001 28923363

[B46] Su Y. P. Ye X. L. Li H. Xu W. Chu K. D. (2020). Simultaneous Determination of Caffeic Acid,protocatechuic Acid and Chlorogenic Acid in Gnaphalium Affine by UPLC-MS/MS. J. Int. Pharm. Res. 47, 1001–1005. 10.13220/j.cnki.jipr.2020.11.017

[B47] Szklarczyk D. Morris J. H. Cook H. Kuhn M. Wyder S. Simonovic M. (2017). The STRING Database in 2017: Quality-Controlled Protein-Protein Association Networks, Made Broadly Accessible. Nucleic Acids Res. 45, D362–D368. 10.1093/nar/gkw937 27924014 PMC5210637

[B48] Tanchuk V. Y. Tanin V. O. Vovk A. I. Poda G. (2016). A New, Improved Hybrid Scoring Function for Molecular Docking and Scoring Based on AutoDock and AutoDock Vina. Chem. Biol. Drug Des. 87, 618–625. 10.1111/cbdd.12697 26643167

[B49] Tao Y. Su D. Li W. Cai B. (2018). Pharmacokinetic Comparisons of Six Components from Raw and Vinegar-Processed *Daphne Genkwa* Aqueous Extracts Following Oral Administration in Rats by Employing UHPLC-MS/MS Approaches. J. Chromatogr. B Analyt Technol. Biomed. Life Sci. 1079, 34–40. 10.1016/j.jchromb.2018.02.005 29428673

[B50] Tian C. Liu X. Chang Y. Wang R. Lv T. Cui C. (2021). Investigation of the Anti-inflammatory and Antioxidant Activities of Luteolin, Kaempferol, Apigenin and Quercetin. South Afr. J. Bot. 137, 257–264. 10.1016/j.sajb.2020.10.022

[B51] Tong C. Xu J. Fu Q. Long R. Peng S. Shi S. (2019). Rapid Extraction, Discrimination and Quantification of Thermally Unstable Isomeric Acteoside and Isoacteoside in Natural Products by Online Extraction-Quadrupole Time-Of-Flight Tandem Mass Spectrometry. Anal. Methods 11, 2148–2154. 10.1039/C8AY02584C

[B52] Vetal S. Bodhankar S. L. Mohan V. Thakurdesai P. A. (2013). Anti-inflammatory and Anti-arthritic Activity of Type-A Procyanidine Polyphenols from Bark of *Cinnamomum Zeylanicum* in Rats. Food Sci. Hum. Wellness 2, 59–67. 10.1016/j.fshw.2013.03.003

[B53] Vogl S. Atanasov A. G. Binder M. Bulusu M. Zehl M. Fakhrudin N. (2013). The Herbal Drug Melampyrum Pratense L. (Koch): Isolation and Identification of its Bioactive Compounds Targeting Mediators of Inflammation. Evid. Based Complement. Alternat Med. 2013, 395316. 10.1155/2013/395316 23533479 PMC3600302

[B54] Wang B. He Y. W. Zhao L. Ji W. P. (2020a). Study on Effect of Aconite Decoction on Anti-inflammation and Joints of Rats with Chronic Infectious Arthritis. Chin. Mod. Doct. 58, 40–44.

[B55] Wang C. F. Xu J. Z. Wang S. Y. Wang Q. H. Yang B. Y. Kuang H. X. (2020c). Study on the Chemical Constituents of Antibacterial Part from Medicated Leaven Massa. Lishizhen Med. Mater. Med. Res. 31, 2350–2353.

[B56] Wang H. Zhou X. M. Wu L. Y. Liu G. J. Xu W. D. Zhang X. S. (2020d). Aucubin Alleviates Oxidative Stress and Inflammation via Nrf2-Mediated Signaling Activity in Experimental Traumatic Brain Injury. J. Neuroinflammation 17, 188. 10.1186/s12974-020-01863-9 32539839 PMC7294631

[B57] Wang R. S. Liu J. Tong P. He S. J. Wu Y. W. Zuo J. P. (2016). Hydroxytriptolide (LLDT-8) Inhibits Inflammatory Cytokines and Promotes Apoptosis in Human Rheumatoid Arthritis Fibroblast-like Synoviocytes by TNF-α Induced via Ras-MAPKs Signaling Pathway. Acta Univ. Tradit. Med. Sinensis Pharmacol. Shanghai. 30, 82–87.

[B58] Wang X. Jiang Z. T. Yan G. J. Shao Z. Y. Feng X. Y. Pan J. H. (2018). Simultaneous Determination of 8 Components in Total Flavanones of Sedum Sarmentosum Bunge by UPLC-MS/MS. Chin. Pharm. 29, 1222–1226.

[B59] Wang X. Y. (2015). Study on Chemical Constituents and Pharmacological Activity of Pholiota Adiposa. Jilin: Jilin Agricultural University.

[B60] Wang Y. Zhou Z. Han M. Zhai J. Han N. Liu Z. (2020b). The Anti-inflammatory Components from the Effective Fraction of Syringae Folium (ESF) and its Mechanism Investigation Based on Network Pharmacology. Bioorg. Chem. 99, 103764. 10.1016/j.bioorg.2020.103764 32222616

[B61] Wang Z. Linghu K. G. Hu Y. Zuo H. Yi H. Xiong S. H. (2019). Deciphering the Pharmacological Mechanisms of the Huayu-Qiangshen-Tongbi Formula through Integrating Network Pharmacology and *In Vitro* Pharmacological Investigation. Front. Pharmacol. 10, 1065. 10.3389/fphar.2019.01065 31607918 PMC6767993

[B62] Wu L. Du S. Yang F. Ni Z. Chen Z. Liu X. (2020). Simultaneous Determination of Nineteen Compounds of Dahuang Zhechong Pill in Rat Plasma by UHPLC-MS/MS and its Application in a Pharmacokinetic Study. J. Chromatogr. B Analyt Technol. Biomed. Life Sci. 1151, 122200. 10.1016/j.jchromb.2020.122200 32526664

[B63] Xia Z. Zhang H. X. Xu T. Q. Chen Y. M. Zhou G. X. (2021). Phenylpropanoids from Fruits of *Xanthium Sibiricum* . Chin. Pharm. J. 56, 13–22. 10.1080/14786419.2020.1806273 32787575

[B64] Xu A. L. Chen Z. Bi X. L. Jiang J. Y. Zhang J. N. Li S. M. (2019). Simultaneous Determination of Five Components in Jasminum Elongatum by UHPLC-MS/MS. J. Guangdong Pharm. Univ. 35, 378–382. 10.16809/j.cnki.2096-3653.2019040101

[B65] Yang J. Q. Li Y. M. Yang Y. F. Wu T. Li X. F. Kang X. D. (2020). Chemical Constituents of *Callicarpa Nudiflora* Leaves. J. Chin. Med. Mater. 1619–1623. 10.13863/j.issn1001-4454.2020.07.015

[B66] Yang L. Meng X. Yu X. Kuang H. (2017). Simultaneous Determination of Anemoside B4, Phellodendrine, Berberine, Palmatine, Obakunone, Esculin, Esculetin in Rat Plasma by UPLC-ESI-MS/MS and its Application to a Comparative Pharmacokinetic Study in normal and Ulcerative Colitis Rats. J. Pharm. Biomed. Anal. 134, 43–52. 10.1016/j.jpba.2016.11.021 27875787

[B67] Yang L. L. Xiao N. Li X. W. Fan Y. Alolga R. N. Sun X. Y. (2016). Pharmacokinetic Comparison between Quercetin and Quercetin 3-*O*-*β*-Glucuronide in Rats by UHPLC-MS/MS. Sci. Rep. 6, 35460. 10.1038/srep35460 27775094 PMC5075792

[B68] Yi J. P. Wu Y. Z. Yu N. Yu Z. W. Xie F. Y. Yuan Q. (2016). VEGF Gene Polymorphisms Affect Serum Protein Levels and Alter Disease Activity and Synovial Lesions in Rheumatoid Arthritis. Med. Sci. Monit. 22, 316–324. 10.12659/MSM.894912 26825024 PMC4750902

[B69] Yin Y. T. Guo Q. X. Chen F. Hong F. (2020). Overview on the Pharmacological Action of Tricin. Guangming J. Chin. Med. 35, 2105–2108. 10.3969/j.issn.1003-8914.2020.13.060

[B70] Zhan J. S. Zhan K. Chen X. L. Huo J. H. Zhao G. Q. (2018). Lipopolysaccharide-induced Effects of Tricin on Inflammatory and Lacto-Protein Gene Expression in Bovine Mammary Epithelial Cells. Prata. Sci. 35, 441–448. 10.11829/j.issn.1001-0629.2017-0184

[B71] Zhang B. Wang X. Li S. (2013a). An Integrative Platform of TCM Network Pharmacology and its Application on a Herbal Formula, Qing-Luo-Yin. Evid. Based Complement. Alternat Med. 2013, 456747. 10.1155/2013/456747 23653662 PMC3638581

[B72] Zhang L. N. Deng R. X. Wang Y. Liu M. M. Liu P. (2019a). Chemical Constituents from Rhizome of *Phlomis Umbrosa* . Chin. Pharm. J. 54, 450–456.

[B73] Zhang M. Y. Zhu Z. M. Yao D. Chen Q. H. Bao X. W. Chen J. W. (2019b). Simultaneous Detection of Cordycepin, D-Mannitol, Adenosine and Inosine in Cordyceps Militaris by HILIC-MS/MS. Chin. J. Bioproc. Eng. 17, 430–436. 10.3969/j.issn.1672-3678.2019.04.015

[B74] Zhang X. Wang L. Zheng Z. Pi Z. Liu Z. Song F. (2017). Online Microdialysis-Ultra Performance Liquid Chromatography-Mass Spectrometry Method for Comparative Pharmacokinetic Investigation on Iridoids from *Gardenia Jasminoides* Ellis in Rats with Different Progressions of Type 2 Diabetic Complications. J. Pharm. Biomed. Anal. 140, 146–154. 10.1016/j.jpba.2017.03.040 28351019

[B75] Zhang Y. Wang K. Chen H. He R. Cai R. Li J. (2018). Anti-inflammatory Lignans and Phenylethanoid Glycosides from the Root of Isodon Ternifolius (D.Don) Kudô. Phytochemistry 153, 36–47. 10.1016/j.phytochem.2018.05.017 29860140

[B76] Zhang Z. J. Sun D. X. Wang J. Dong L. H. Guo P. P. Wang C. Y. (2013b). Simultaneous Determination of Eight Components in Kouyanqing Granules by LC-MS/MS. Chin. Tradit. Pat. Med. 35, 952–956. 10.3969/j.issn.1001-1528.2013.05.020

[B77] Zhao Y. S. Bi Y. Q. Lei L. J. Zhu X. H. Lv Y. Zhang C. H. (2017). [Variety Systematization and Research Progress of Mongolian Medicine "Bashaga"]. Zhongguo Zhong Yao Za Zhi 42, 998–1004. 10.19540/j.cnki.cjcmm.20170121.027 28994546

[B78] Zheng C. Wang J. Liu J. Pei M. Huang C. Wang Y. (2014). System-level Multi-Target Drug Discovery from Natural Products with Applications to Cardiovascular Diseases. Mol. Divers. 18, 621–635. 10.1007/s11030-014-9521-y 24792224

[B79] Zhou H. Z. Luo W. Chen S. Chen H. L. Li G. Y. Li L. M. (2021). Coumarins from Flowers of *Stellera Chamaejasme* and Their Biological Activities. Chin. Tradit. Herb. Drugs 52, 943–950. 10.7501/j.issn.0253-2670.2021.04.006

[B80] Zhou J. Wang Q. Xiang Z. Tong Q. Pan J. Wan L. (2019). Network Pharmacology Analysis of Traditional Chinese Medicine Formula *Xiao Ke Yin Shui* Treating Type 2 Diabetes Mellitus. Evid. Based Complement. Alternat Med. 2019, 1–15. 10.1155/2019/4202563 PMC6754917 31583009

[B81] Zhou W. Wang Y. Lu A. Zhang G. (2016). Systems Pharmacology in Small Molecular Drug Discovery. Int. J. Mol. Sci. 17, 246. 10.3390/ijms17020246 26901192 PMC4783977

